# Den Kalten Krieg neu denken? Neue Studien zum Kalten Krieg

**DOI:** 10.1007/s42520-022-00441-y

**Published:** 2022-05-23

**Authors:** Kirsten Bönker

**Affiliations:** Historisches Institut, Abteilung für Osteuropäische Geschichte, Universität zu Köln, Köln, Deutschland

**Keywords:** Kalter Krieg, Ost-West-Konflikt, Kultur- und Verflechtungsgeschichte, Cultural Diplomacy, Soft Power, Cold War, East-West-Conflict, Cultural and Entangled History, Cultural Diplomacy, Soft Power

## Abstract

Neuere Studien dekonstruieren die Binarität des Kalten Krieges mit globalen, transnationalen und kulturgeschichtlichen Perspektiven. Sie zeigen, dass bestimmte Interpretationsrahmen wie Interdependenz, Konvergenz der Systeme, Globalisierung oder Dekolonisierung bereits von Zeitgenoss_innen mit dem Narrativ des Kalten Krieges verbunden wurden. Dies bereichert das Bild der innenpolitischen Positionen und außenpolitischen Interessen, die im Westen gegenüber der Sowjetunion vertreten worden sind. Neuere Forschungen zeigen aber auch, dass es an wirtschafts- oder mediengeschichtlichen Studien mangelt, die sich auf beide Seiten des Eisernen Vorhangs konzentrieren.

## Abstract (en)

Using global, transnational, and cultural-historical perspectives, recent studies deconstruct the binarity of the Cold War. The studies show that certain frames of interpretation, such as interdependence, convergence of systems, globalisation or decolonisation, had already been combined with the narrative of the Cold War by contemporaries. This enriches the picture of domestic positions and foreign policy interests of the West vis-à-vis the Soviet Union. However, recent research also shows that there is a lack of studies in economic or media history that focus on both sides of the Iron Curtain.

Ist der Kalte Krieg zurück oder war er nie wirklich beendet? Diese Frage bewegt die westeuropäische Öffentlichkeit spätestens seit der russischen Annexion der Krim im Frühjahr 2014.^1^ Mit der neuerlichen Zeitenwende am 24. Februar 2022, als Vladimir V. Putin die russländische Armee in die Ukraine einmarschieren ließ, hat diese Frage nicht nur eine neue bittere und realpolitische Relevanz gewonnen. Rückblickend erscheinen nun der Giftanschlag auf Aleksej A. Naval’nyj, die Hackerangriffe auf den Deutschen Bundestag oder die *fake news*, mit denen russische Trolle seit Jahren den propagandistischen Wettbewerb mit ‚dem Westen‘ in den sozialen Medien anheizen, als Teil der russischen Strategie, sich vom ‚Westen‘ zu distanzieren. Damit aber nicht genug, denn Medien, wie der mittlerweile verbotene TV-Sender „Russia Today“, versuchten zugleich, westeuropäische Demokratien zu destabilisieren. Vor diesem Hintergrund betrachtete die Frankfurter Allgemeine Zeitung den russischen Impfstoff gegen COVID-19 als „ein Instrument des hybriden Krieges“.^2^

Bereits vor dem Krieg verwies der Rückgriff in Medien und Politik auf das überholt geglaubte Ordnungsmuster des Kalten Krieges auf die anhaltende Deutungsmacht des Ost-West-Konflikts, der nun mit Wucht zurückgekehrt ist.^3^ Seine historisch-kulturelle Dimension scheint in Putins Hass auf die freiheitlich-demokratischen Ordnungen des Westens auf und ist Teil der perfiden Rechtfertigung des Krieges geworden. Der russländische Patriotismus legitimiert das Putin-Regime innenpolitisch schon seit Jahren und basiert auf einer angeblich überlegenen russländischen Kultur. Der Patriotismus lebt auch davon, dass alte Feindbilder über ‚den Westen‘ seit Langem wieder befeuert und neue konstruiert werden.^4^ Damit knüpft das Putin-Regime an die ideologisch-kulturelle Konkurrenz an, die die politischen Blöcke bereits im Kalten Krieg mit Praktiken der *cultural diplomacy* und der *soft power* ausgetragen haben. So war den Zeitgenoss_innen des Kalten Krieges klar, dass dieser trotz der Rüstung und Stellvertreterkriege nicht zuletzt ein „Superpower War of Words“^5^ war. Es war das amerikanische Journal „Newsweek“, das den Ost-West-Konflikt just in der heiklen Rüstungsphase Ende 1983 als einen ideologischen und medialen Wettbewerb um die ‚Herzen und Köpfe‘ der Menschen in aller Welt beschrieb. Die Forschung hat diese kulturelle Dimension des Systemwettbewerbs lange missachtet, sie aber in den letzten Jahren deutlich verstärkt in den Blick genommen. Diesen Forschungstrend aufnehmend, werden im Folgenden neue Studien zum Kalten Krieg aus den Jahren 2019 und 2020 aus einer kultur- und verflechtungsgeschichtlichen Perspektive besprochen. Zunächst wird daher das aktuelle Forschungsfeld umrissen, um die Analysefolie und den Bewertungsmaßstab für die folgenden Ausführungen zu verdeutlichen und Fragen anhand der neueren Forschungen zu entwickeln.

## Der Kalte Krieg in der neueren Forschung: Von binär zu multipolar, verflochten, (trans-)national und global

Neuere Studien haben den Kalten Krieg als ein Gewebe beschrieben, das mit seiner ideologischen Lagerbildung nicht nur in die internationalen Beziehungen, sondern in nahezu alle gesellschaftlichen Bereiche hineinwirkte. Der Konflikt betraf somit die allermeisten Menschen auf die eine oder andere Weise auch im Alltag. Die Blockkonfrontation erzeugte ein binäres Denken und eine politische Sprache der Abgrenzung. Der Systemwettbewerb berührte so unterschiedliche Aspekte wie die nukleare Aufrüstung, das *space race*, die Menschenrechte, den globalen Handel, die Rohstoffversorgung, die Zirkulation von Medien, Informationen und Wissen, internationale Sportveranstaltungen oder den Umweltschutz. Er beeinflusste die Prozesse der Dekolonialisierung und der Globalisierung entweder unmittelbar oder nahm deren Einflüsse auf, denn der Kalte Krieg war zumeist Teil, wenn auch nicht unbedingt die Ursache dieser Prozesse. Trotz der Stellvertreterkriege, die in aller Welt geführt wurden, wurde der Kalte Krieg schon 1990 vereinzelt als ein „imaginärer“ Konflikt analysiert.^6^

2010 kam Gottfried Niedhart in einer Bestandsaufnahme zu Neuerscheinungen zum Kalten Krieg zu dem Schluss, dass die Forschung mittlerweile ein breites Themenspektrum als „internationale Geschichte“ abdecke. Zwar überwogen vor etwa zehn Jahren politikgeschichtliche Arbeiten, die sich auf Staat und Diplomatie konzentrierten, noch deutlich, doch zunehmend rückten auch Gesellschaft und Ökonomie in den Blick.^7^ Weiterhin haben politikgeschichtliche Arbeiten Relevanz und Nachfrage, sodass nach wie vor zahlreiche Studien erscheinen, die sich mit staatlichen Akteuren befassen und eher militär-, diplomatie- und politikgeschichtliche Höhenkammperspektiven einnehmen. Zugleich hat sich aber die Tendenz, den Kalten Krieg mit kultur-, gesellschafts- und verflechtungsgeschichtlichen Ansätzen zu untersuchen, seit Niedharts Bestandsaufnahme erheblich verstärkt.^8^ Bemerkenswert ist zudem die weitere Ausdifferenzierung der Ansätze, Methoden und Themen, sodass die Forschungsliteratur allein der letzten zehn Jahre kaum mehr zu überschauen ist. Da nun nicht zuletzt die imaginären, metaphorischen, visuellen und emotionalen Dimensionen des Kalten Krieges in den Blick gerückt sind, hat die Forschung in den letzten Jahren immer deutlicher gezeigt, wie die Blockkonfrontation spezifische Zugehörigkeiten, Ängste und Feindbilder erzeugte.^9^

Weiter hat sich der Blick auf den Kalten Krieg geografisch erheblich geweitet. Wurden lange vor allem die Supermächte und ihr außenpolitisches Agieren betrachtet, brechen neuere Studien das bipolare Konfliktnarrativ auf, indem sie Staaten und Gesellschaften jenseits der Supermächte einbeziehen, Verflechtungen, Aneignungen und Transfers analysieren. Durch eine solche Dezentralisierung des Kalten Krieges werden auch seine globalen Aus- und Rückwirkungen betrachtet. China rückt als einflussreiche Kraft des globalen Konflikts in den Blick. Die sich dekolonisierenden Staaten erhalten in dieser Forschung *agency*, eigene Interessen und Strategien.^10^ Viele Studien haben die globalen Dimensionen der Konkurrenz um die sich dekolonisierenden Staaten naheliegenderweise zunächst in militär- und/oder politikgeschichtlichen Perspektiven behandelt.^11^ Doch auch hier sind kultur- und verflechtungsgeschichtliche Ansätze immer wichtiger geworden, um zu untersuchen, wie Ost und West neben den wirtschaftlichen, technologischen und militärischen Hilfeleistungen zudem mit kulturellen Angeboten um die jungen postkolonialen Staaten warben. Dabei widmen sich Studien zunehmend der Frage, wie die Sowjetunion im *Global South* mit Mitteln der Kulturdiplomatie und *soft power* um Einfluss kämpfte.^12^

Kultur- und Globalgeschichte haben demnach das Interesse für die ideologisch-kulturelle Wirkung der Blockkonfrontation gestärkt.^13^ Um den Kalten Krieg als alltäglichen und einflussreichen Deutungsrahmen zu analysieren, richtet sich der Blick daher nun auch stärker nach innen auf die Gesellschaften und damit auf die innenpolitischen Resonanzen der friedlichen Konkurrenz um das bessere Gesellschaftssystem. Das Interesse gilt oft transnationalen Austauschprozessen. Auf diese Weise rücken neue Akteursgruppen in den Blick, die als potenzielle Brückenbauer und Grenzgängerinnen über den Eisernen Vorhang hinweg wirkten: Dies waren Frauen und Männer aus verschiedenen Gruppen, wie Journalist_innen, Wirtschaftsvertreter_innen, Wissenschaftler_innen, Studierende, Kunstschaffende, Sportler_innen, Tourist_innen.^14^ Neben den Konfrontationen und Abgrenzungen betrachten die Studien damit auch Annäherungen und Verflechtungen.^15^

Das Forschungsfeld zum Kalten Krieg ist also vielfältig, dynamisch und bietet zahlreiche Anknüpfungspunkte wie offene Fragen. Vor diesem Hintergrund werden die methodischen Ansätze der zu besprechenden Studien beleuchtet: Reflektieren sie den Kalten Krieg als Ordnungs- und Deutungsschema? Wird er als eigene Epoche oder als spezifischer Abschnitt in einem längeren Ost-West-Konflikt im 20. Jahrhundert gedeutet? Nutzen die Studien ihn als kulturgeschichtlichen Analyserahmen, indem Strategien der *soft power* und *cultural diplomacy* analysiert werden?

Außerdem soll verfolgt werden, inwiefern die Studien einerseits die Binarität des Kalten Krieges dekonstruieren und globale beziehungsweise transnationale Perspektiven aufwerfen. Welchen Platz räumen sie andererseits Osteuropa – der Sowjetunion, aber auch den ostmitteleuropäischen Staaten als eigenständige Akteure – im globalen Kalten Krieg ein? Dazu zählt auch die grundsätzliche Frage nach Konvergenzen, Wechselwirkungen und Austausch zwischen Ost und West: Werden beide Seiten des Eisernen Vorhangs miteinander in Bezug gesetzt? Wann dominieren nationale Perspektiven beziehungsweise wann wird der Kalte Krieg multiperspektivisch erzählt, sodass neben den Supermächten die globalen Dimensionen durch lokale beziehungsweise regionale Akteur_innen, Prozesse und Auswirkungen sichtbar werden? Welche Themen werden überhaupt untersucht? Inwiefern erweitern oder dekonstruieren die aktuellen Untersuchungen klassische Erklärungsansätze, die auf ideologische Abgrenzung, binäre Deutungsmuster und das Verhältnis zwischen Zentrum und Peripherie schauen, Phasen der Hoch- und Abrüstung, der Krisen und Entspannung in den Blick nehmen oder mit dem Primat der Politik das Ringen um Macht und das Handeln ‚großer Männer und Frauen‘ in den Vordergrund rücken?

Die meisten der hier zu besprechenden Studien führen den Kalten Krieg im Titel. Der zweite Abschnitt diskutiert die Bücher, die den Kalten Krieg vorrangig als Epochenzuschreibung verstehen und einen politikgeschichtlichen Zugriff wählen. Von Interesse ist, wie sie den Zeitabschnitt konstruieren und ihn empirisch füllen. Werfen sie neue Perspektiven auf den Beginn und das Ende des Kalten Krieges? Verändert der Blick auf geopolitische Räume jenseits von Europa unser Bild vom Kalten Krieg als Epoche?

Während das dritte Kapitel zwei Studien vorstellt, die den Kalten Krieg in einer wirtschaftsgeschichtlichen Perspektive betrachten, rückt der vierte Abschnitt kultur- und verflechtungsgeschichtliche Untersuchungen in den Mittelpunkt. Von Interesse ist, wie sie den Kalten Krieg als Analyserahmen nutzen. Wie machen sie den Systemwettbewerb in seiner Verflochtenheit und Wechselseitigkeit konzeptionell fruchtbar? Im Fazit und Ausblick wird die Frage aufgegriffen, wie sich der Eiserne Vorhang methodisch überbrücken und der Kalte Krieg als eine transnationale Kultur- und/oder Verflechtungsgeschichte erzählen lässt, indem Konvergenzen, Zirkulationen und Abgrenzungen von Akteur_innen, Praktiken, Ideen und Deutungen untersucht werden.

## Der Kalte Krieg als Epoche: Wie begann er und wodurch endete er?

Der Kalte Krieg wird oft als eine vergangene politische Konstellation in einer abgeschlossenen Epoche betrachtet. Allerdings erweisen sich der Beginn und das Ende des Kalten Krieges als nicht so eindeutig, wie es Jahreszahlen und Daten suggerieren. Odd Arne Westad schlägt in seiner monumentalen Darstellung zur „Weltgeschichte“ des Kalten Krieges vor, dass der Konflikt als Konfrontation zwischen Kapitalismus und Sozialismus zwischen 1945 und 1989 seinen Höchststand erreichte.^16^ Auf diesen Zeitrahmen können sich vermutlich viele Historiker_innen einigen. Auch die russischen Publikationen der letzten zwei Jahrzehnte betrachten den Kalten Krieg zumeist ab 1945.^17^ Der Kalte Krieg ist eine zeitgenössische Konstruktion und damit auch ein Quellenbegriff, aus dem ein scheinbar selbsterklärender politischer Metabegriff geworden ist. Doch selbst der Kalte Krieg hatte weder zeitgenössisch noch retrospektiv eine universelle Bedeutung. Auch zeichnete er als Deutungsmuster, wie es George Orwell im Oktober 1945 mit Blick auf die politischen Folgen der Atombombe für das zukünftige Mächtegleichgewicht verwendete, trotz aller ideologischen Spannungen keine teleologische Entwicklung vor. Unlängst hat Norman Naimark den Beginn des Kalten Krieges aus einer europäischen Perspektive analysiert und nachgezeichnet, dass die Teilung Europas 1945 keinesfalls bereits besiegelt war. Vielmehr kommt Naimark zu dem Schluss, dass Stalin keinen konkreten Plan verfolgt und entsprechend pragmatisch, zum Teil kompromissbereit agiert habe, stets darauf bedacht, einen neuerlichen Krieg in Europa zu vermeiden. Dies bedeutete, wie Naimark argumentiert, dass die geopolitische Situation bis Ende 1947 trotz der aufkommenden Spannungen deutlich offener war, als bisher angenommen. Allerdings – und das ist nach wie vor eine der zentralen offenen Fragen für die Frühphase des Kalten Krieges – lassen sich bis heute, wie Naimark betont, nur Vermutungen über die Motive Stalins anstellen.^18^ Insofern erweist sich ein solcher Perspektivwechsel, wie ihn Naimark mit sieben Länderfallstudien anbietet, als fruchtbar, um Stalins Interessen auf die Spur zu kommen. Auf diese Weise lässt sich der Übergang zur Systemkonfrontation als politischen Aushandlungsprozess mit zahlreichen Akteur_innen und als einen Kampf um Worte, Ideen und Repräsentationen erkennen. Die Zeitgenoss_innen fochten um kollektive Repräsentationen, sie markierten die Systemkonfrontation sprachlich und zeitlich als eine neue Weltordnung gegenüber anderen Phasen. Auf westlicher Seite verorteten sie sich mit den politischen Schlagworten des Kalten Krieges^19^ und des Eisernen Vorhangs^20^ in dem seit der Russländischen Revolution schwelenden Ost-West-Konflikt. So ließen sich Feindbilder der Zwischenkriegszeit wiederbeleben, die fortan eine politische Sprache der Abgrenzung prägten. Insofern ist zu erwarten, dass neue Studien, nicht zuletzt, indem sie über Europa hinausblicken, auch neue Aspekte zum Entstehen des Kalten Krieges beitragen. Das lässt sich etwa bei der Arbeit von Thomas K. Robb, Historiker an der Oxford Brookes University, und David Gill, Politologe an der University of Nottingham, beobachten. Sie haben für den frühen Kalten Krieg von 1945 bis 1955 untersucht, wie Australien und Neuseeland in Kooperation und Auseinandersetzung mit den traditionellen Großmächten USA und Großbritannien nach neuen diplomatischen Strategien im asiatisch-pazifischen Raum suchten.^21^ Zwar diskutieren die Autoren in ihrer Studie „Divided Allies. Strategic Cooperation Against the Communist Threat in the Asia-Pacific During the Early Cold War“ keine konkreten Ereignisse als Beginn des Kalten Kriegs. Doch ihre Analyse der außenpolitischen Strategien und Interessen der vier Staaten zum Kriegsende 1945 verdeutlicht in einer globalen Perspektive den fließenden Übergang vom Zweiten Weltkrieg, der im asiatisch-pazifischen Raum ohnehin länger andauerte als in Europa, zum Kalten Krieg. So wurden hier spätestens mit der Kapitulation Japans am 15. August 1945 die Machtverhältnisse mit einer anderen Dynamik als in Europa und mit divergierenden Interessen neu ausgehandelt. Dabei ging es zum einen um die Frage, ob Großbritannien seine koloniale Vorherrschaft wiederherstellen könnte oder ob sich die USA mit ihrer Forderung durchsetzen würden, den europäischen Kolonien den Weg in die Souveränität zu ermöglichen (S. 12). Zum anderen zeichnete sich bereits ab, dass die Sowjetunion und auch China größeren Einfluss in dieser Region beanspruchten.

Die Studie ist interdisziplinär angelegt, indem sie einen klassisch diplomatiegeschichtlichen Zugriff mit dem politikwissenschaftlichen Ansatz der Internationalen Beziehungen verbindet. Dementsprechend betrachten die Verfasser staatliche Interessen und außenpolitisches Handeln. Dazu rücken sie drei Abkommen in den Mittelpunkt, die die Sicherheit im asiatisch-pazifischen Raum garantieren sollten (ANZUS, SEACDT und SEATO). Mit der Einschränkung, dass ihre Darstellung eine rein westliche Perspektive auf den Kalten Krieg im asiatisch-pazifischen Raum wirft, zeichnen die beiden Autoren überzeugend nach, wie zunächst die enge Allianz nach dem Sieg über den gemeinsamen Gegner Japan zerbrach, um sich dann angesichts der wahrgenommenen kommunistischen Bedrohung im Kalten Krieg neu zu arrangieren.

Bereichernd ist, wie die Autoren anhand der vier Staaten die machtpolitischen Dynamiken und imaginierten Bedrohungsszenarien jenseits von Europa und Afrika verfolgen, und damit den vergleichsweise weniger betrachteten asiatisch-pazifischen Raum in eine globale Geschichte des Kalten Krieges einbeziehen. Aufschlussreich sind die unterschiedlichen, oft wirtschaftlich motivierten Interessenlagen der Staaten, die quer zu den ideologisch-politischen Spannungen des noch jungen europäischen Kalten Krieges lagen. So entwickelten auch die USA und Großbritannien teilweise widersprechende Interessen und geopolitische Strategien, weil sie die Bedrohungsszenarien im asiatisch-pazifischen Raum unterschiedlich hierarchisierten. Entsprechend gab es Differenzen zwischen Großbritannien und den USA um die diplomatische, aber auch handelspolitische Vorherrschaft in Asien. Dabei sahen die Vereinigten Staaten Japan seit den frühen 1950er Jahren eher als strategischen Partner an. Australien und Neuseeland hielten im Gegensatz dazu ein wiedererstarkendes Japan für eine größere Bedrohung als die Sowjetunion und China. Zugleich hatten sie ein grundsätzliches Interesse an prosperierenden Handelsbeziehungen, vor allem mit China. Robb und Gill analysieren, wie solche inneren und querliegenden Interessenkonflikte mitunter eine gemeinsame harmonische Allianz gegen die kommunistischen Mächte konterkarierten. Zukünftige Studien sollten diese politischen Bruchlinien in der – im weitesten Sinne – westlichen Welt, die hier für das erste Nachkriegsjahrzehnt aufgezeigt worden sind, im weiteren Verlauf des Kalten Krieges verfolgen. Zudem müssen auch die Beziehungen zu den Staaten des sowjetischen Blocks und zu China einbezogen werden und diese als Akteure mit eigenständigen Strategien und Interessen in den Blick rücken.

Der frühe Kalte Krieg steht auch in weiteren Neuerscheinungen im Mittelpunkt, die zudem die Zwischenkriegszeit einbeziehen, aber ganz andere Erkenntnisinteressen formulieren. So beansprucht der amerikanische Politikwissenschaftler Kyong-Min Son bei seiner Untersuchung der Transformation der Demokratietheorie im Kalten Krieg, einen ideengeschichtlichen Zugang mit der normativen politischen Theorie zu verbinden (S. 12). Sein Ausgangspunkt ist die gegenwärtige Krise der Demokratie – deren Ort er allerdings nicht spezifiziert. Sie werfe die Frage auf, wie der Neoliberalismus die moderne Demokratie praktisch und konzeptionell herausgefordert habe. Außerdem interessiert sich der Autor für die inneren Schwächen, die die Demokratie anfällig gemacht haben könnten (S. 2–4). Als ursächlich betrachtet er Entwicklungen der praktizierten Demokratie und der Demokratietheorie in den Nachkriegsjahren, als die Erfahrungen mit diktatorischen Regimen den Umgang mit demokratischen Verfahrensweisen ebenso erheblich beeinflussten wie die demokratietheoretischen Diskussionen. Sein spezifisches Augenmerk gilt dabei den konzeptionellen Rollen des *demos* und der politischen Institutionen in den Theorien (S. 4–6). Inwiefern die Idee der Demokratie in der Nachkriegszeit einen allgemeinen Wandel erlebt habe (S. 11), diskutiert Son anhand von Autor_innen wie Hannah Arendt, Norbert Wiener oder Friedrich Hayek. Diese Fragen, die hier allerdings durch den politikwissenschaftlichen Ansatz recht normativ und mit dem alleinigen Fokus auf Theorien und Ideen untersucht werden, sind relevant, um die Akteur_innen und Argumente in den Diskussionen der Nachkriegsjahre über die Demokratie in größeren intellektuellen, politischen und kulturellen Zusammenhängen verorten zu können. Angesichts des weltweiten ideologischen Systemwettbewerbs, in dem auch die sozialistischen Staaten des sogenannten Ostblocks den Begriff der Demokratie im Sinne einer Volksdemokratie für sich reklamierten, übte die praktische und theoretische Resilienz der Demokratien liberaler Provenienz eine besondere Strahlkraft im Wettbewerb um die ‚Herzen und Köpfe‘ der Menschen in aller Welt aus.

Originell ist, wie Son den Kalten Krieg zeitlich definiert und den Untersuchungszeitraum seiner Studie fasst: Er unterscheidet zwischen einem historisch-politischen Verständnis des Kalten Krieges, demzufolge er 1947 mit der Verkündung der Truman-Doktrin begonnen hätte, und dem Kalten Krieg als „intellectual event“. Als solches hätte er einen längeren Vorlauf gehabt und reiche bis in die Mitte der 1930er Jahre zurück. Daher beginnt Sons Untersuchung zu diesem Zeitpunkt und endet in den späten 1970er Jahren, als der Neoliberalismus an Einfluss gewann (S. 13). Leider begründet der Autor insbesondere den Beginn seines Untersuchungszeitraumes nicht. Dennoch verweisen diese Überlegungen zumindest darauf, dass die Periodisierung des Kalten Krieges nicht so eindeutig ist, wie die Systemkonkurrenz der Supermächte nach dem Zweiten Weltkrieg lange suggerierte. In den leicht differierenden Lesarten dauerte der Kalte Krieg von 1945 beziehungsweise 1947 bis zum Fall der Berliner Mauer 1989 oder längstens bis zum Zerfall der Sowjetunion 1991. Als der ideologische Konflikt zwischen Kapitalismus und Sozialismus beendet war, schien auch der Kalte Krieg Geschichte. Seine Periodisierung hebt damit auf die Blockkonfrontation und ihrem Changieren zwischen Krise und Détente ab. Ohne den Gedanken auszuführen, spricht Son zu Recht an, dass die „intellectual underpinnings“ des Kalten Krieges älter als die Truman-Doktrin und schon Mitte der 1930er Jahre angelegt gewesen seien. Son betrachtet den Kalten Krieg damit als eine spezifische Phase eines länger andauernden Ost-West-Konflikts. Dieser historische Kontext aber interessiert Son nicht, sodass er die Begriffe „Cold War“ und „postwar“ irritierenderweise synonym verwendet (S. 13). Die Unterscheidung zwischen einem politischen und einem intellektuellen Ereignis wird der Vielschichtigkeit des Kalten Krieges nicht gerecht. Historiker_innen haben, wie eingangs angedeutet und im Folgenden noch weiter ausgeführt werden wird, schon anschaulich herausgearbeitet, dass der Kalte Krieg mit seinen politischen und kulturellen Dimensionen auf beiden Seiten des Eisernen Vorhanges bis tief in den Alltag der Menschen gedrungen ist. Son reduziert die Konkurrenz weitgehend auf die Frage, wie die von ihm betrachteten Demokratietheoretiker Kapitalismus und Demokratie in Einklang brachten, wie demzufolge Wohlstand und demokratische Mitsprache verteilt werden sollten und welche Rolle dabei der Antikommunismus spielte. Indem der Markt eine wachsende Bedeutung als Regulativ erlangte, die staatliche Regulierung der Finanzmärkte abnahm und öffentliche Ausgaben gekürzt wurden, ohne dass dies intellektuellen Protest für mehr Partizipation nach sich zog, gewannen neoliberale Praktiken an Bedeutung. Um den Neoliberalismus zurückzudrängen, stellten aber laut Son die Demokratie – präziser: die Theorien über die Demokratie – der Nachkriegszeit keine Alternative dar (S. 180f.).

Der Kalte Krieg im Sinne der ideologischen Frontstellung zwischen den Systemen ließe sich mit diesen überwiegend ideengeschichtlichen Fragen verbinden. Für Son aber hat er keine konzeptionelle Funktion, sondern bleibt nur eine zeitliche Zuschreibung für die Jahre nach dem Ende der nationalsozialistischen Diktatur. Dennoch ist auch aus geschichtswissenschaftlicher Sicht eine längere Perspektive auf ideengeschichtliche Entwicklungen von Demokratie und Neoliberalismus aufschlussreich. Allerdings kontextualisiert Son den Einfluss der europäischen Diktaturen, die er unreflektiert unter dem Begriff „Totalitarismus“ zusammenfasst, leider weder praxeologisch noch begriffsgeschichtlich. Der Totalitarismus hätte, wie der Autor bekräftigt, tiefe Spuren in der Demokratietheorie nach dem Zweiten Weltkrieg hinterlassen. Das ist eine interessante Behauptung, zumal Son als eine Folge die unterschwellige Angst der Demokratietheoretiker_innen vor der ‚ignoranten Masse‘ anführt, während gleichzeitig die Vorstellung, dass die Wissenschaft Mensch und Gesellschaft – im Sinne der ‚Massen‘ – in verschiedener Hinsicht leiten könnte beziehungsweise sollte, erheblich an Einfluss gewonnen habe (S. 11f.). Inwiefern diese Vorstellungen tatsächlich Einfluss auf politische Praktiken genommen haben, verfolgt Son aber leider im Verlauf seiner Darstellung nicht weiter. Aus geschichtswissenschaftlicher Sicht fehlt seiner ideengeschichtlichen Diskussion daher an vielen Stellen eine historische Kontextualisierung sowie eine kultur- und diskursanalytische Verortung, die auch Praktiken in den Blick nimmt: In welchen Diskursen des Kalten Krieges sind die demokratietheoretischen Diskussionen zu verorten? Inwiefern wurden Grenzen des Sagbaren verschoben? In welchen soziokulturellen und politischen Kontexten beanspruchten die Sprecher_innen Deutungsmacht? Welche handlungsleitenden Wirkungen erzeugten die Äußerungen? Solche Fragen, die Historiker_innen stellen würden, liegen nicht im Erkenntnisinteresse des Verfassers.

Ähnlich wie Son rückt auch Guido Thiemeyer, Professor für Neuere Geschichte in Düsseldorf, in seiner Einführung „Totalitarismus und Kalter Krieg“ den Systemwettbewerb mit einer bislang ungewöhnlichen zeitlichen Perspektive in einen originellen argumentativen Zusammenhang. Thiemeyer setzt 1920 ein und betont damit die Bedeutung eines Ost-West-Konflikts für die europäische Geschichte, der sich demzufolge seit der Russländischen Revolution von 1917 und der Gründung der Sowjetunion 1922 entwickelte. Während es damit naheliegend erscheint, bestimmte Phänomene des Kalten Krieges in der längeren Perspektive eines Ost-West-Konflikts mit all seinen Brüchen und (Dis‑)Kontinuitäten zu untersuchen, überrascht 1970 als Endpunkt einer Einführung zum Kalten Krieg. Zwar sind zeitliche Zäsuren und Periodisierungen immer Konstruktionen, in diesem Fall sind sie aber ausschließlich westeuropäisch geprägt. Sie ergeben sich zudem nicht aus dem Systemwettbewerb, sondern aus westeuropäischen Phänomenen, wie dem Übergang zur Dienstleistungsgesellschaft Ende der 1960er Jahre (S. 151). Das ist schade, denn das Bändchen ist in der Reihe „Europäische Geschichte der Neuzeit“ erschienen. ‚Osteuropa‘ und der Kalte Krieg als ideologischer Wettbewerb spielen aber kaum eine konzeptionelle und die Darstellung strukturierende Rolle. Osteuropa wird lediglich additiv und untergeordnet beziehungsweise als ‚das Andere‘ präsentiert.

Ein zentrales Argument für die Periodisierung der Einführung lautet zum Beispiel die Herausbildung des „modernen Interventionsstaates“ (S. 46f.). Der wurde in der Sowjetunion aber schon in den 1920ern unter Anwendung massiver Repressionen in einer extremen Form implementiert, sodass ein solches Merkmal im europäischen Vergleich deutlich differenziert werden müsste, um für gesellschaftsgeschichtliche Entwicklungen aussagekräftig zu sein. Eher noch könnte man auf bestimmte Steuerungsmechanismen, wie den Wirtschaftsplan, abheben. (S. 23f.). Andere Begründungen für den Betrachtungszeitraum erscheinen bei aller in einer Einführung notwendigen Zuspitzung holzschnittartig bis irreführend: So fasst Thiemeyer unter anderem zusammen, dass sich in allen westeuropäischen Staaten seit 1920 und in den osteuropäischen Staaten nach 1990 – was außerhalb des Betrachtungszeitraumes liegt – ein demokratisch verfasster Rechtsstaat „in zum Teil gewaltsamer Auseinandersetzung mit autoritären Diktaturen“ durchgesetzt habe. Auch seien in ganz Europa strukturell ähnliche Parteiensysteme entstanden, die aber nationale Besonderheiten aufgewiesen hätten (S. 46f). Was bedeutet diese These zum Beispiel mit Blick auf die KPdSU oder die französische PCF im Vergleich zu den westdeutschen Parteien oder den Blockparteien der DDR?

Generell wäre eine schärfere Begrifflichkeit wünschenswert gewesen. Erstaunlicherweise diskutiert Thiemeyer den so prominent in den Titel gesetzten Begriff des Totalitarismus nicht einmal in seiner Ambivalenz als Kind des Kalten Krieges, sondern nennt nur eine sehr traditionelle Definition (S. 39f.). Der Autor verwendet „autoritär“ und „totalitär“ deckungsgleich, sodass er gesellschaftliche, politische oder wirtschaftliche Entwicklungen nur unpräzise erfasst. Auch in einer Einführung ist es unerlässlich, die Definitionen der Begriffe gegeneinander abzuwägen und auf ihre wissenschaftsgeschichtliche Entwicklung hinzuweisen (etwa S. 38–47). Thiemeyer aber arbeitet mit ungewöhnlichen Begriffsschöpfungen. Er spricht von einem „autoritären Sozialismus“ (S. 17), von „autoritären Diktaturen“ (S. 47) – gibt es auch nicht-autoritäre Diktaturen? –, „totalitären Diktaturen bis 1945“ (S. 62) oder „autoritärer Planwirtschaft“ (S. 79).

Für die osteuropäischen Länder bezieht Thiemeyer wiederholt die DDR oder die Sowjetunion ein. Leider tragen diese Einlassungen wenig zu einer konzeptionellen Sicht auf eine europäische Geschichte bei. Dies liegt nicht zuletzt daran, dass der Autor einige Punkte nicht genügend einordnet, dass Zusammenhänge und Begriffe schief oder sachlich falsch sind. So wirken seine Ausführungen zur Eingabepraxis in der DDR im Verhältnis zur westlichen Verwaltungsgerichtsbarkeit wenig informiert und auch für eine knappe Einführung unverständlich verkürzt. Statt auf dem Wege der Verwaltungsgerichtsbarkeit hätten sich, so Thiemeyer, „die Bürger durch so genannte ‚Eingaben‘ bei der Verwaltung an der Verbesserung des Staates und seiner Administration beteiligen“ können (S. 61). Wenn es nicht wie eine (n)ostalgische Aussage eines ehemaligen DDR-Funktionärs klingen soll, hätte die Eingabepraxis mindestens knapp im Kontext der politischen Kommunikation in einem autoritären Regime eingeordnet werden müssen.

Im Kapitel zur Gewalt als innenpolitisches Phänomen in der Zwischenkriegszeit betrachtet Thiemeyer die Russländische Revolution und den Bürgerkrieg (S. 113–115). Nicht nur an dieser Stelle spricht er ärgerlicherweise von „Bolschewisten“ (S. 33), das doch als Signalwort der LTI in wissenschaftlichen Darstellungen nicht verwendet werden sollte. Immerhin benutzt er auf derselben Seite einmal den korrekten Begriff „Bolschewiki“ (wissenschaftlich: Bol’ševiki), übersetzt ihn aber fälschlich mit „Die Roten“ (S. 114). Sie hätten im Bürgerkrieg „gegen die anderen politischen Parteien (Sozialdemokraten, Konservative: ‚Die Weißen‘)“ gekämpft. Es ist hier nicht der Platz, um die Fehler und die schiefe Argumentation geradezurücken, aber auch die Bol’ševiki waren Sozialdemokraten, während es keine Partei gab, die sich „Konservative“ nannte. Auch kämpften im Bürgerkrieg keine „Parteien“. Deutsche und k.u.k. Verbände besetzten nicht nach dem Frieden von Brest-Litovsk im März 1918 „große Teile des ehemaligen Zarenreiches handstreichartig“ (S. 114), sondern waren bereits zuvor unter anderem ins Baltikum, nach Weißrussland und in die Ukraine weit nach Osten vorgerückt. Der Verlust an Bevölkerung, Wirtschaftskraft und Territorium war für Russland erheblich, aber es waren angesichts der Ausdehnung bis zum Pazifik keinesfalls „große Teile“ des ehemaligen Zarenreiches (S. 114). Und schließlich wurde der KGB nicht in den 1920er Jahren (S. 114), sondern erst 1954 gegründet.

Abschließend belegt das Kapitel über Konsum nach 1945, dass der Autor den Kalten Krieg als Systemwettbewerb nicht konzeptionell nutzt, um eine gemeinsame, verflochtene Geschichte Europas zu schreiben. Konsum wird hier nicht als zentrales Feld der Konkurrenz analysiert, sondern allein in nationalen Perspektiven. So dient der Kalte Krieg der Einführung insgesamt lediglich als plakative zeitliche Zuschreibung. Die Chance, einen Erkenntnisgewinn daraus zu ziehen, den Begriff auf der Basis neuerer Forschungen als konzeptionelle Klammer für ein miteinander verflochtenes Konkurrenzverhältnis in Europa zu nutzen, wird so vertan.

Dass sich Zäsuren je nach Perspektive wandeln, verdeutlicht auch die Studie des britischen Politologen Archie Brown, emeritierter Professor an der Oxford University. In der umfangreichen, klassisch politikgeschichtlichen Darstellung „The Human Factor. Gorbachev, Reagan, and Thatcher, and the End of the Cold War“^22^ beleuchtet er nun bereits mit seiner dritten Studie erneut das Ende des Kalten Krieges.^23^ Ziel ist es, nicht nur das Wesen des Konflikts der Supermächte zu erklären, sondern insbesondere die Art und Weise, wie er beendet wurde: „What exactly *was* the Cold War and why did it end in the way it did *when* it dit?“ [Hervorh. jeweils im Orig.] (S. 2). Enden lässt Brown den Kalten Krieg mit dem Zerfall der Sowjetunion und dem Rücktritt Michail S. Gorbačevs als Präsident im Dezember 1991. Damit greift er – in allerdings konventioneller Vorgehensweise – den aktuellen Trend der Zeitgeschichte auf, die sich nach dem Boom der Studien zu den 1970er Jahren nun zunehmend der Perestrojka und der Befriedung des Systemwettbewerbs zuwendet. Wer einen konzisen und gut informierenden Überblick über biografische Perspektiven verbunden mit einem politikgeschichtlichen Abriss wichtiger Ereignisse des Kalten Krieges seit den späten 1970er Jahren sucht, kann dieses Buch zur Hand nehmen.

Eloquent erzählt Brown das Ende des Kalten Krieges als eine diplomatisch-menschliche Leistung zweier ‚großer Männer‘ – Michail S. Gorbačev und Ronald Reagan – und einer Frau, Margaret Thatcher. Nachdem der Verfasser bereits 1996 Gorbačev als ehemaligen Generalsekretär der KPdSU in den Mittelpunkt einer Untersuchung zur Perestrojka gestellt hat, rückt er erneut die Frage nach politischer Führung und politischem Wandel in den Mittelpunkt.^24^ Dabei knüpft der Autor an seine Bewertung von 1996 an, denn schon damals lautete sein Argument, dass Gorbačev den größten Teil dazu beigetragen habe, den Kalten Krieg friedlich und eben zu jenem Zeitpunkt zu beenden, als er zu Ende ging. Sein neues Buch ist ähnlich faktenorientiert und kopflastig, wobei viele Aspekte nicht zuletzt aus seinen eigenen Studien gut bekannt sind. So zeichnet er die Lebenswege der drei Akteur_innen und wichtige realpolitische Zusammenhänge konventionell mit Blick auf Ereignisse nach. Grundsätzlich blendet er gesellschaftliche Entwicklungen, die nennenswerten Anteil am Zerfall der Sowjetunion und dem Machtverlust Gorbačevs hatten, aus.

Dennoch ergänzt Brown mit den biografischen Ausführungen seine eigene Argumentation, die er bereits für Gorbačev in „Seven Years that Changed the World“ aufgezeigt hat: Nun führt er auch das jeweilige soziokulturelle Umfeld der drei Personen an, das ihre politischen Vorstellungen prägte. So zeichnet er Gorbačevs Werdegang und Gedankenwelt differenzierter als zuvor, nicht zuletzt, weil er von der neu erschienenen Biografie William Taubmans profitiert.^25^

Den „menschlichen Faktor“, den Brown so prominent im Titel setzt, verdeutlicht er ein weiteres Mal anhand der persönlichen Beziehungen der ‚großen Drei‘. Diese Beziehungen spielten bereits in seinem letzten Buch eine zentrale Rolle, in dem er den politischen Willen Gorbačevs und Reagans unterstreicht, mit dem Wettrüsten auch den Kalten Krieg zu beenden. Diese These weitet Brown nun aus, indem er stärker noch den Einfluss Thatchers betont, die er hier nahezu als den Mittelpunkt der Beziehungen zeichnet. Bereits Ende 1983, als noch nicht abzusehen war, dass Gorbačev Generalsekretär der KPdSU werden könnte, waren sich Reagan und Thatcher einig, dass es an der Zeit sei, „normale Beziehungen“ zur Sowjetunion aufzubauen: „We will have to deal with the Soviet Union. But we must deal with it not as we would like it to be, but as it is. We live on the same planet and we have to go on sharing it“ (S. 120). Diese neue Haltung erleichterte die Kommunikation mit Gorbačev zusätzlich, der rasch Zuspruch in Großbritannien erhielt. Brown zeichnet Margaret Thatcher als die stärkste Unterstützerin unter den westlichen Regierungschefs, die Gorbačevs Reformpolitik und seine Bemühungen um nukleare Abrüstung nachhaltig befürwortete (S. 190). So versuchte Thatcher, Reagan in seiner Annäherungspolitik an die Sowjetunion zu bestärken (S. 222). Andersherum suchte Reagan selbst den steten Austausch mit Thatcher über die Sowjetunionpolitik. So machte er zum Beispiel auf seiner Rückkehr aus Moskau im Juni 1988 in London Station (S. 232). Thatcher wiederum traf Gorbačev wiederholt zum persönlichen Gespräch und stand in regem Briefverkehr mit ihm.

Indem Brown die Personen in den Mittelpunkt seiner Darstellung rückt, erzählt er das Ende des Kalten Krieges anhand ihrer Treffen, ihres Austausches und nicht zuletzt auch der bereits anderweitig ausführlich behandelten Abrüstungsverhandlungen zwischen den westlichen Regierungsspitzen und Gorbačev. Immerhin thematisiert Brown auch das jeweilige heimische Diskussionsumfeld, in das die transnationalen Beziehungen eingebettet waren. So wurden überall Stimmen laut, die die Annäherungen zwischen der Sowjetunion und dem Westen kritisch sahen. Einhergehend geriet auch Gorbačevs Perestrojka innenpolitisch zunehmend unter Beschuss durch konservative Kräfte. Reagan und US-Außenminister George P. Shultz mussten sich mit Abrüstungsgegnern, die den Abbau von Atomwaffen ablehnten, auseinandersetzen. Allerdings war die Denuklearisierung, wie sie das INF-Abkommen festlegte, auch im Westen nicht unumstritten. Während Thatcher nur zähneknirschend akzeptierte, dass damit auch der britische Bestand an Atomwaffen infrage gestellt wurde, bezeichnete der französische Verteidigungsminister André Giraud die Verhandlungen im Vorfeld in einer eigenwilligen Parallele als „nukleares München“. Gleichzeitig lehnten Thatcher und François Mitterand beide das amerikanische SDI-Programm ab (S. 223). Auch der amerikanische Vize-Präsident George H. W. Bush gehörte zu denjenigen, die die Annäherungen an die Sowjetunion skeptisch betrachteten, und noch nach Reagans Rückkehr aus Moskau versicherten: „The Cold War isn’t over“ (S. 233). Konservative Stimmen, wie Richard Nixon und Henry Kissinger, bezichtigten Reagan wiederholt der Naivität und zu großen Nachgiebigkeit im Umgang mit der Sowjetunion (S. 307).

Die Skepsis zielte nicht zuletzt auf die vermuteten ‚wahren‘ Interessen Gorbačevs, den einige für undurchschaubar und daher für eine Gefahr für den Westen hielten. Der spätere Sicherheitsberater von George Bush, Brent Scowcroft, bescheinigte Gorbačev: „[He] was trying to kill us with kindness“. Gorbačev sage Dinge, die „wir hören wollten“ und mache „zahlreiche verlockende Vorschläge“, um die „propagandistische Überlegenheit im Kampf um die internationale öffentliche Meinung“ zu gewinnen (S. 233). Eigentlich wolle Gorbačev aber lediglich das sowjetische System dynamisieren, um wettbewerbsfähiger zu werden. Daher sei er für den Westen gefährlicher als sein Vorgänger (ebd.).

Diese zeitgenössischen Auseinandersetzungen sind ebenso bekannt, wie das Argument unstrittig sein dürfte, dass auch die westlichen Regierungschefs ihren persönlichen Anteil am friedlichen Ende des Kalten Krieges hatten. George Bush wäre noch zu nennen, der hier aber wie auch der französische Präsident François Mitterand und Bundeskanzler Helmut Kohl kaum eine Rolle spielt. Es liegt jedenfalls nahe, die Prägungen, Ansichten und Praktiken der ‚großen‘ Männer und Frauen einzubeziehen, wie es Brown tut. Allerdings liegen ähnliche Studien zu diesen Personen bereits vor, auf die sich Brown zum Teil stützt, aber nicht über sie hinausgeht.^26^

Lesenswert ist aber, dass Brown das politische Siegernarrativ vieler Angehöriger der Reagan-Administration, die betonen, dass die amerikanische Militärkraft und die ideologische Unnachgiebigkeit die Sowjetunion in die Knie gezwungen hätten, dezidiert zurückweist (S. 288). Seine Erklärung, wie und warum der Kalte Krieg ausgerechnet zu dem gegebenen Zeitpunkt endete, zielt allerdings letztlich nicht auf die Bemühungen der westlichen Regierungschefs – so sehr er diese auch für „signifikant“ hält (S. 290). Vielmehr betrachtet er als den bei Weitem wichtigsten Grund den „transformativen Wandel“, der in der Sowjetunion stattgefunden habe (ebd.). Doch auch den verortet er allein auf höchster politischer Ebene und betont die Rolle Gorbačevs: Ohne ihn sei die Liberalisierung und Öffnung nicht denkbar gewesen. Das ist sicher richtig, doch überliefert Brown innenpolitisch ein eher weichgezeichnetes Bild Gorbačevs, bei dem seine Fehler und Fehleinschätzungen, wie das Festhalten an Lenin’schen Idealen, der Umstand, dass er die Sprengkraft der Nationalitätenfrage übersah und die Existenz des Zusatzprotokolls des Hitler-Stalin-Pakts wider besseres Wissen leugnete, keine Rolle spielen. Stattdessen habe er gegen starke Widerstände die außenpolitische Öffnung durchgesetzt, denn Befürworter eines politischen Pluralismus seien selbst unter den „reformorientierten *meždunarodniki*“ – also den sowjetischen Expert_innen für die Weltregionen und internationalen Beziehungen – kaum zu finden gewesen beziehungsweise diese hätten sich einen solchen Wandel nicht vorstellen können (S. 294f.). Dieses Argument, dass das Ende der Sowjetunion für die allermeisten Menschen in der Sowjetunion nicht vorstellbar gewesen sei, erinnert stark an den häufig zitierten Buchtitel Alexei Yurchaks „Everything was Forever Until It Was No More“, ohne dass Brown darauf Bezug nähme, geschweige denn – abgesehen von Taubmans Biografie – andere geschichtswissenschaftliche Forschung zur Kenntnis nimmt.^27^ Die sowjetische Gesellschaft und ihr Beitrag zum Ende des Kalten Krieges interessieren Brown nicht, obwohl er selbst einleitend auf den – man ist geneigt zu sagen – Irrglauben und Überlegenheitsdünkel vieler westlicher, vor allem amerikanischer Beobachter verweist, dass die Perestrojka das sowjetische Eingeständnis der eigenen Unterlegenheit angesichts des amerikanischen wirtschaftlichen und militärischen Drucks gewesen sei (S. 2). Diese These wäre es tatsächlich wert, sie auch in einer verflechtungsgeschichtlichen Perspektive zu untersuchen und zu fragen, wie der Kalte Krieg in den 1980er Jahren als Teil des Legitimitätsglaubens an das sowjetische Regime in der gesellschaftlichen Wahrnehmung an Bedeutung verlor. Die Frage, wie sich Feindbilder auflösten und Praktiken der Annäherung an den Westen in der Gesellschaft durchsetzten, ist gerade angesichts der aktuell wieder gegenteiligen Ausprägung sehr relevant. Der Krieg gegen die Ukraine zeigt, dass das Putin-Regime nicht nur das sowjetische Imperium wiederherstellen möchte, sondern innenpolitisch problemlos an alte Feindbilder gegenüber dem Westen anknüpfen kann.

Brown wirft die spannende und wichtige Frage auf, in wessen Interesse der politisch-ökonomische Wandel in der Sowjetunion gewesen sei (S. 295). Schließlich bedeutete politische Pluralisierung für die Angehörigen des Militärs, des KGB und anderer in seiner Sicht „nichts als Ärger“ (S. 296). Browns Antwort bleibt unbefriedigend, denn er sucht den Grund bei Gorbačev, das heißt in der „fundamental significance of power and authority which accrued to the general secretary“ (S. 297).

Da Brown selbst bekundet, dass die eigentlich spannenden Prozesse in der Sowjetunion zu untersuchen wären, wirkt sein Ansatz methodisch und analytisch umso mehr aus der Zeit gefallen: „The liberalization and evolving democratization of the Soviet political system, accompanied by the new freedom of speech, contributed greatly to the growth of international trust“ (S. 290). Es waren allerdings die ‚normalen‘ Sowjetbürger_innen, die diese neuen Möglichkeiten ergriffen und ihren Teil zur Transformation beitrugen. Diese Prozesse und Praktiken entdeckt die Forschung aber gerade erst. Daher bestimmen bislang die zeitgenössischen Diagnosen der Politolog_innen oder Journalist_innen und die oft retrospektiven Erzählungen der Regierungsvertreter_innen die Narrative und müssen nun als das, was sie sind, dekonstruiert werden: als zu historisierende und zu kontextualisierende Erzählungen der zeitgenössischen Akteur_innen. Dass die westlichen Beobachter_innen viele der gesellschaftlichen Entwicklungen nicht angemessen nachvollziehen und bewerten konnten, belegen Browns Ausführungen zu den zeitgenössischen Diskussionen. So lässt er wiederholt seine eigene Zeitzeugenschaft einfließen, da er zusammen mit anderen Sowjetunion-Spezialisten als Berater Thatchers fungierte. Wenig überraschend hat auch von den britischen Experten, die im September 1983 zu einem Seminar eingeladen wurden, niemand den grundlegenden Umbruch des sowjetischen Systems vorausgesehen (S. 114f.). Wissen und Prognosen über die Sowjetunion waren immer Bereiche, in denen verschiedene Expert_innen um die Aufmerksamkeit der Politik und der Medien konkurrierten. Brown verdeutlicht dies, indem er sich selbst dafür lobt, dass er in seinem Beitrag zum erwähnten Seminar – anders als die Mitarbeiter des *Foreign Office* – Gorbačev als zukünftigen Generalsekretär der KPdSU vorausgesehen habe (S. 117). Diese Passagen, in denen es um den Politikwechsel Thatchers hin zu einem aktiven und kommunikativen Stil gegenüber der Sowjetunion geht, sind spannend zu lesen, unterstreichen aber die Notwendigkeit, sie zu historisieren und in eine europäisch-globale Verflechtungsgeschichte der Perestrojka und Transformation einzuordnen. Brown selbst belässt es bei einer Betrachtung der persönlichen Netzwerke und Interaktionen auf höchster politischer Ebene, um das friedliche Ende des Kalten Krieges zu erklären.

## Energie, Handel und Waffen im Kalten Krieg: Wirtschaftsinteressen zwischen Entspannung und Abgrenzung

Anhand der französischen und italienischen Ölkonzerne untersucht Roberto Cantoni in einer lehrreichen Studie, wie Fragen der Energiesicherheit nicht nur zu technisch-wissenschaftlichen Innovationen und neuem Wissen im Bereich der Seismologie, der Geophysik und des Klimawandels führten, sondern auch nationale Sicherheitsinteressen und die transnationalen Beziehungen zwischen 1944 und 1962 beeinflussten.^28^ Italienische Politiker nutzten die Neustrukturierung des italienischen Ölkonzerns AGIP unter staatlicher Ägide ab 1944/45, um die Versuche der USA und Großbritanniens abzuwehren, Kontrolle über den italienischen Energie- und Rohstoffmarkt zu gewinnen. So begannen sie mithilfe von Geowissenschaftler_innen und Energietechniker_innen bereits vor Kriegsende die Weichen für ihre zukünftige spezifische Machtposition im Ost-West-Konflikt zu stellen, indem sie erfolgreich den Zugang zu Expert_innenwissen über Energieversorgung und Infrastrukturen sicherten und die Kontrolle über eine Schlüsselindustrie behielten. Italien und Frankreich strebten jeweils eine eigenständige, nicht selten gegeneinander gerichtete Energiepolitik an, die sie unabhängig von der Blockkonfrontation und anderen westlichen Staaten machen sollte. So versuchte Frankreich, die neu erschlossenen Öl- und Gasvorkommen in Algerien und Zentralafrika dem Zugriff insbesondere britischer und amerikanischer Firmen zu entziehen, um zusammen mit der Entwicklung eines eigenen Atomprogramms eine eigenständige Position im Konflikt der Supermächte zu erlangen. Nicht unähnlich pochte auch Italien auf nationale Unabhängigkeit in Fragen der Energiesicherheit und -politik, während die USA den Marshall-Plan als Hebel nutzten, um den amerikanischen Energiekonzernen Zugang zum westeuropäischen Markt zu sichern.

Cantoni zeigt die Dissonanzen im westlichen Block aber nicht nur zwischen Westeuropa und den USA, sondern auch anhand der Konkurrenz zwischen Frankreich und Italien. In Algerien unterstützte nämlich die italienische ENI, der Nachfolgekonzern der AGIP, die algerischen Nationalisten gegen die französische Kolonialmacht. 1957 schloss ENI Abkommen mit dem Iran und Ägypten und stach dort die britischen und amerikanischen Konkurrenten aus. Als besonderen und von den westlichen Partnern kritisch beäugten Schachzug aber nahm ENI 1958 Verhandlungen mit der Sowjetunion auf, um einen Öl-für-Technik-Deal abzuschließen. Ab 1960 importierte Italien in großen Mengen sowjetisches Öl und lieferte dafür die Ausrüstung für neue sowjetische Pipelines. Aus Angst, die Sowjetunion könne ihre Machtposition in Europa über die Kontrolle des Ölmarktes weiter ausbauen, initiierten die USA daraufhin ab 1962 ein NATO-Embargo auf den Export von Röhrentechnologie. Es war allerdings nicht von langer Dauer, da es insbesondere den Handelsinteressen Frankreichs und der Bundesrepublik entgegenstand, die bereits seit den 1950er Jahren Handelsverträge mit der Sowjetunion anstrebten. Divergierende wirtschaftliche und energiepolitische Interessen sorgten also bereits im frühen Kalten Krieg für nennenswerte Bruchstellen im westlichen ‚Block‘ und selbstständiges Verhandeln mit dem ideologischen Gegner. Cantoni zeigt eindrücklich, wie die jeweiligen nationalen Energie- und Sicherheitsinteressen Frankreichs und Italiens Dynamiken erzeugten, die dem binären Blockdenken zuwiderliefen und grundsätzlich ein kohärentes ‚westliches‘ Verhältnis zur Sowjetunion auf der einen Seite und eine gemeinsame europäische Haltung zu den USA auf der anderen Seite infrage stellten. Dabei rückt der Verfasser auch sonst eher weniger beachtete Akteure wie die Algerische Befreiungsfront (FLN) in den Vordergrund, die sehr wohl die Konkurrenz um Energiesicherheit zu ihren eigenen Gunsten ausnutzte. Cantoni versucht, auch die Interessen der Sowjetunion zu berücksichtigen, kann dies jedoch vermutlich wegen mangelnder Sprachkenntnisse nur im Spiegel der westlichen Perspektiven und Annahmen leisten. Gleichwohl ergänzt Cantoni mit seinen westeuropäischen Fallstudien einen Sammelband und eine Dissertation, die ebenfalls 2017 erschienen sind, aber die, anders als er, die sowjetischen Energiestrategien analysieren. Diese Werke verdeutlichen, dass für die Sowjetunion – im Gegensatz zu den USA, aber ähnlich wie für Italien und Frankreich – ideologische Motive regelmäßig hinter wirtschaftspolitischen und geostrategischen Interessen zurückstanden.^29^ Diese Erkenntnis legt nahe, dass Phasen der Entspannung und der Krise abseits der Rüstungspolitik und der Diplomatie und je nach beteiligten Akteur_innen deutlich vielschichtiger und dynamischer verliefen, als die übliche Periodisierung des Kalten Krieges nahelegt, die sich am Verhältnis der Supermächte orientiert.^30^

Da Erdöl und Erdgas als wichtige sowjetische Exportgüter eine zentrale Rolle in den außenwirtschaftlichen Beziehungen der Sowjetunion erlangten, liegt die Relevanz dieses Themenfeldes für ökonomische Dynamiken und politische Beziehungen im Kalten Krieg auf der Hand. Dass hier im diplomatischen Kontext politische und wirtschaftliche Interessen auf beiden Seiten des Eisernen Vorhangs stets neu verhandelt werden mussten, wirkte wiederum insofern auf innenpolitische Prozesse in der Sowjetunion zurück, als der Devisenfluss durch die Öl- und Gasexporte sehr wahrscheinlich den Reformstau der sowjetischen Wirtschaft bis zur Perestrojka überdeckte. Hier gilt es, mit verflechtungsgeschichtlichen Ansätzen die These Stephen Kotkins zu untermauern beziehungsweise herauszufordern, dass die Sowjetunion maßgeblich mithilfe der Rohstoffexporte und damit auch dank der auf Profit und technischen Austausch orientierten Handelsbeziehungen zu den westeuropäischen Staaten in den 1970er Jahren so stabil blieb.^31^

Alleine im Bereich der Energiesicherheit im Ost-West-Konflikt wäre also noch reichlich Potenzial für eine Verflechtungsgeschichte, die zudem den Nord-Süd-Konflikt ausleuchtet und dabei neben sowjetischen auch chinesische Interessen berücksichtigt. Ebenso lohnte es sich, die zentralen Akteure, nämlich die Energieexperten mit ihren Vorstellungen, zu untersuchen, um mehr über ihre Wissensproduktion, ihre Netzwerkpraktiken und politischen Interessen zu erfahren. Auch die ökonomischen Interessen bestimmter Akteursgruppen, die nur scheinbar hinter den Sorgen um Energiesicherheit zurücktraten und die nicht erst beim französischen und westdeutschen Protest gegen das Röhrenembargo wirkten, könnten präziser als Triebkraft und Faktor in den internationalen Beziehungen analysiert werden.

Allgemein stellen die transnationale Wirtschaftsgeschichte und Fragen der politischen Ökonomie im Kalten Krieg, wie sie Cantoni thematisiert, noch ein erstaunlich wenig beachtetes Forschungsfeld dar. Die Frage, in welchen Fällen ökonomische Interessen über ideologische dominierten und die binäre Denklogik infrage stellten, ist eine, die noch großes Potenzial für weitere Forschungen insbesondere mit vergleichenden und verflechtungsgeschichtlichen Ansätzen bietet. Für die Sowjetunion hat Oscar Sanchez-Sibony in einer Pionierstudie aufgezeigt, wie durchlässig der Eiserne Vorhang für nicht-ideologiegetriebene Wirtschaftskontakte war, sodass die Sowjetunion seit den 1920er Jahren immer auch aktiv auf dem globalen Markt agierte. Sanchez-Sibony argumentiert zudem, dass die Sowjetunion keinesfalls einen kommunistischen Kreuzzug im *Global South* unternahm, sondern die sich dekolonisierenden Staaten häufig selbst Wirtschaftskontakte zur UdSSR aufnahmen. Seine Hauptthese lautet daher, dass ökonomische Logiken die ideologischen Motive im sowjetischen Handeln im globalen Kontext häufig ausstachen.^32^

Dass das Wechselspiel zwischen dem Primat der Außenpolitik und den ökonomischen Interessen transnationale Beziehungen im Kalten Krieg erheblich beeinflusste, hat bereits Karsten Rudolph mit einer anschaulichen Studie zur Industrie in den bundesdeutsch-sowjetischen Wirtschaftsbeziehungen unterstrichen.^33^ Seine These, dass Industrievertreter, wie Berthold Beitz oder Otto Wolff von Amerongen zu bundesrepublikanischen Wirtschaftsdiplomaten wurden und auf der Suche nach neuen Absatzmärkten der Neuen Ostpolitik quasi den Weg bereiteten, ist überzeugend. Was fehlt, sind wie so oft die sowjetische Seite und die Vergleichsstudien für andere westeuropäische Länder.

Vor diesem Hintergrund ist es zunächst vielversprechend, dass Stephan Kieninger in seiner 2018 erschienenen Studie „The Diplomacy of Détente. Cooperative Security Policies from Helmut Schmidt to George Shultz“ mit dem Osthandel ebenfalls die ökonomische Dimension der Détente in den Mittelpunkt rückt.^34^ Sein Interesse gilt der Frage, wie über langfristige Handelsbeziehungen gegenseitiges Vertrauen aufgebaut wurde, das dazu beigetragen hat, die Entspannung trotz militärischer Krisen zu verstetigen. Kieninger argumentiert allerdings traditionell diplomatie- und politikgeschichtlich und betrachtet das Handeln ‚großer Männer‘, nämlich vorrangig der im Titel so prominent erwähnten Helmut Schmidt und George Shultz. Insbesondere geht es ihm darum, die konzeptionellen Ansichten Helmut Schmidts über die Bedeutung der Wirtschaftsbeziehungen für die Entspannungs- und Außenpolitik der sozialliberalen Regierung zu analysieren. Dazu hat er vor allem den Nachlass von Helmut Schmidt ausgewertet, zieht aber auch die Papiere Zbigniew Brzezińskis, Margaret Thatchers und Ronald Reagans heran, um die widerläufigen Interessen zwischen wirtschaftlichen und ideologischen Logiken im ‚westlichen‘ Lager aufzuzeigen. Die Administrationen Carter und Reagan betrachteten die westdeutsche Ostpolitik unter dem Motto „Wandel durch Handel“ nämlich höchst kritisch. Schmidt gelang es aber, die bundesdeutsche Strategie auch dann noch zu verteidigen, als die Sowjetunion im Dezember 1979 in Afghanistan einmarschiert war, die Bundesrepublik mit den USA die Olympischen Spiele in Moskau boykottiert und Polen im Dezember 1981 das Kriegsrecht verhängt hatte. Kieninger zeichnet Schmidt dabei – hart an der Grenze zur Hagiografie – als den weitsichtigen Architekten der Entspannungspolitik. Während Hans-Dietrich Genscher, der sowohl in der sozialliberalen als auch in der christliberalen Koalition als Außenminister für Kontinuität in der Ostpolitik stand, erstaunlicherweise kaum Erwähnung findet, habe Schmidt zudem den amerikanischen Außenminister George P. Shultz und Präsident Ronald Reagan von der hohen Bedeutung persönlicher Kontakte zur sowjetischen Führung überzeugt. Neben der exzeptionellen Rolle Schmidts betont Kieninger wenig originell, dass der Kalte Krieg durch Kooperation, Verständigung und Entspannung zum Ende kam.

Mag der Verfasser auch einzelne Aspekte des Schmidt’schen Denkens neu gewichtet haben, so bietet das Buch doch insgesamt keine neuen Erkenntnisse. Weder macht Kieninger sich die Mühe, die westdeutschen Wirtschaftsstrategien – und damit auch das Agieren Schmidts – im Osthandel in längere Kontinuitätslinien einzuordnen noch lässt sich ernsthaft behaupten, dass der Osthandel „under-researched“ (S. 1) sei oder ein „hidden factor“ (S. 2) in den deutsch-sowjetischen Beziehungen gewesen sei. Bedauernswert ist, dass ökonomische Interessen und auch die Energiepolitik, die Kieninger anspricht, nicht an gesellschaftliche und kulturelle Prozesse rückgebunden werden. Vor allem aber erzählt der Autor eine rein westliche Geschichte, die die Interessen und Initiativen der Sowjetunion höchstens erahnen lässt. Insofern ist auch die These von der durch Schmidt orchestrierten Entspannung, die den Kalten Krieg beendet habe, eindimensional und wirft kein neues Licht auf das Ende des Ost-West-Konflikts. Die Studie bietet hauptsächlich Leser_innen, die keine deutschsprachige Forschung verfolgen, einen konzisen Einblick in den Osthandel und die westdeutsche Entspannungspolitik der Ära Schmidt.

So unbestritten die Rolle des Osthandels für die deutsch-sowjetischen Beziehungen ist, so wenig ist über die Unternehmens- und Marketingstrategien der westdeutschen Rüstungsindustrie bekannt. Sie setzte aus ökonomischen Eigeninteressen einen deutlichen Kontrapunkt zur Neuen Ostpolitik, indem sie Abgrenzung betonte und die Sowjetunion als Feindbild zur politischen Legitimation ihrer ökonomischen Interessen brauchte. Die Mannheimer Wirtschaftshistorikerin Stefanie van de Kerkhof untersucht das aufschlussreiche Thema der Marketingstrategien von Rüstungsunternehmen und verortet es zeitlich mit der Gründung der Bundesrepublik 1949 bis zur Wiedervereinigung 1990.^35^

Es liegt auf der Hand, dass „Waffen und Sicherheit“ nicht nur Machtfaktoren im Ost-West-Konflikt waren. Als Symbole für Frieden und Bedrohung prägten sie auch Diskursmuster in der ideologischen Auseinandersetzung, erzeugten Imaginationen und Emotionen, die bis in den Alltag der Menschen drangen und manche zum Handeln antrieben. So trug in vielen westlichen Ländern die Angst vor einem Atomschlag zum zivilgesellschaftlichen Engagement der Friedens‑, Umwelt- und Anti-Atomkraftbewegungen bei. Die Frage, wie Waffen und Sicherheit nicht nur innenpolitisch und diplomatisch zusammengedacht, sondern auch marktstrategisch und öffentlichkeitswirksam, wie van de Kerkhofs Untertitel anspricht, beworben wurden, ist also überaus relevant für eine Geschichte des Kalten Krieges und des Systemwettbewerbs. Van de Kerkhof zeichnet in ihrer umfangreichen, quellengesättigten Studie detailliert den Wiederaufstieg der bundesdeutschen Rüstungsindustrie zum drittgrößten Exporteur der Welt nach. Dabei rückt die Autorin die öffentlichen Selbstrepräsentationen verschiedener Rüstungsunternehmen mit einem marketing- und unternehmensgeschichtlichen Zugriff in den Mittelpunkt. Mit dem Begriff der Public Relations, verstanden als „Beeinflussung der Öffentlichkeit oder relevanter Teilöffentlichkeiten durch Selbstdarstellung“ (S. 11), analysiert sie die Produkt‑, Programm‑, Kommunikations- und Distributionspolitik der Rheinmetall GmbH und von Krauss-Maffei.

Die westdeutsche Rüstungsindustrie war nach dem Zweiten Weltkrieg moralisch und wirtschaftlich am Boden, doch die Blockbildung im Kalten Krieg, die Gründung der NATO und die zunehmenden Stellvertreterkriege verschafften ihr neue Absatzmärkte im In- und Ausland. Die Rüstungsindustrie stand mit ihren hochpolitischen Produkten wenig überraschend besonders im Fokus der medialen Öffentlichkeit. Exporte wurden skandalisiert, Vergabepraktiken für öffentliche Aufträge standen nicht selten unter Korruptionsverdacht – diese Phänomene sind bis heute gut bekannt und unterstreichen die Bedeutung ihrer historischen Erforschung. Im Kontext der Blockkonfrontation war die bundesdeutsche Rüstungsindustrie, so ein wichtiges Ergebnis der Studie, zunehmend bestrebt, ihre öffentliche Außendarstellung und Reputation zu verbessern. Eine zentrale Werbestrategie zielte daher darauf, das Vertrauen der Bundesbürger_innen zu gewinnen. Die Werbebotschaft bestand darin, dass die Unternehmen angesichts der nuklearen Bedrohung mit ihren Waffen ‚Sicherheit‘ produzierten. Weiter vermieden es die Unternehmen, Exportgeschäfte öffentlich zu thematisieren und schrieben sie selbst in der internen Kommunikation mit ihren Mitarbeiter_innen klein. Neben dem Einsatz von Werbekampagnen sprachen die PR-Abteilungen gezielt bestimmte gesellschaftliche Gruppen und Teilöffentlichkeiten, wie Journalisten, Politiker, Wehrtechnik-Interessierte oder Schüler und Studenten als zukünftige Wehrdienstleistende, an, um sie als Multiplikatoren ihrer Anliegen zu gewinnen. So erhielten Schülerzeitungen Anzeigeaufträge oder Beiträge über Ausbildungsgänge in den Unternehmen. Journalisten wurden zu Hintergrundgesprächen beim Sektfrühstück eingeladen oder erhielten Aufmerksamkeiten zu Feiertagen. Die Grenze zur Korruption dürfte nicht selten überschritten worden sein (S. 253–273).

Eine ikonografische Analyse der Werbematerialien zeigt, dass die Unternehmen modern, sauber, technologisch innovativ, sozial verantwortlich und vertrauenswürdig wirken wollten, während Krieg und Gewalt – wenig überraschend – nicht abgebildet wurden. Die modernen Waffen sollten aber nicht nur das Image der Unternehmen aufwerten, sondern zugleich staatstragend die technische Überlegenheit der Bundesrepublik repräsentieren. Die PR-Abteilungen konstruierten das Bild, dass militärische Stärke in der Blockkonfrontation friedenssichernde Macht bedeute. „Sicherheit“ stellte laut der Autorin den Hauptdiskursstrang dar, der in den 1970er und 1980er Jahren in Varianten wie „Sicherheit des Friedens“ oder „Sicherheit der Freiheit“ vermittelt wurde. Dieser Diskurs ließ sich leicht mit anderen, wie dem der Abschreckung verknüpfen, aber auch auf zivile Produkte, zum Beispiel von Rheinmetall, beziehen. Auf diese Weise versprach das Unternehmen, die Welt auch durch Stoßdämpfer sicherer zu machen und ließ ‚Sicherheit‘ als eine technologische und messbare Leistung darstellen. Dies gipfelte in dem Slogan „Sichere Waffen für einen sicheren Frieden“ (S. 398–433).

Innenpolitisch sah sich die Rüstungsindustrie zunehmend durch die Kritik der Gewerkschaften, der Friedens- und Anti-Atomkraftbewegungen herausgefordert und somit einem steigenden Druck der öffentlichen Meinung ausgesetzt (S. 279–284). Außenpolitisch schien der Kalte Krieg als Bedrohungsszenario so omnipräsent, dass er innenpolitisch die zentrale Legitimationsressource der Rüstungsindustrie darstellen konnte. Die Autorin deutet an, dass die Kommunikations- und Unternehmensstrategien auf außenpolitische Entwicklungen in der Blockkonfrontation reagierten. Angesichts der Entspannungspolitik der späten 1960er, frühen 1970er Jahre ließ Rheinmetall den nicht-militärischen Sektor restrukturieren. Der Fall der Berliner Mauer, den die Verfasserin als Ende des Ost-West-Konflikts betont, bewirkte, dass sich die Außendarstellung des Unternehmens stärker auf die zivilen Produktionssparten ausrichtete (Kapitel 3). Trotz dieser offensichtlichen Anknüpfungspunkte nutzt die Autorin den Kalten Krieg kaum als Analyserahmen, um das Handeln der Akteur_innen oder die Diskurse des Rüstungsmarketings zu deuten, die sie in Kapitel 4 untersucht. Seltsamerweise legt sie mehrfach unser retrospektives Wissen vom nahenden „Ende“ des Kalten Krieges als Maßstab an, um die Strategien der Rüstungswerbung oder Aussagen der Medien um 1985 herum zu interpretieren (z. B. S. 305f., S. 466f.).^36^ Hier wäre es spannend gewesen, zum einen die zeitgenössischen Deutungsmuster der Akteur_innen zu untersuchen und zu fragen, inwiefern der Kalte Krieg möglicherweise tatsächlich an Bindekraft verloren hatte. Das hieße, ihn als Quellenbegriff zu kontextualisieren. Zum anderen wäre zu prüfen, welche gesellschaftlich relevanten Feindbilder, wie Antikommunismus oder die Angst vor ‚den Russen‘, die Entscheidungen der Rüstungsakteure geprägt haben. Dazu zählen auch Wahrnehmungen, Emotionen und Imaginationen, wie Angst vor der nuklearen Katastrophe, die Last der deutschen Kriegsschuld, das Streben nach einer deutschen Wiedervereinigung oder gar nach den verlorenen deutschen Ostgebieten oder – im Gegenteil – der Wunsch nach Entspannung und Verständigung. Solche Deutungsmuster beeinflussten auch die Einordnung des Systemkonflikts, sodass das Rüstungsmarketing auf sie reagieren musste. Allerdings spielt der Kalte Krieg als binäres Ordnungsschema, das die Praktiken der Rüstungsmanager und PR-Abteilungen rahmte, für die Autorin keine explizite Rolle. Das ist zwar legitim, aber dennoch schade, denn sie nimmt neben diesen Akteuren auch noch andere Gruppen wie Politiker, Journalisten oder Friedensaktivisten in den Blick, die allesamt dazu beitrugen, den Kalten Krieg überhaupt als Wettbewerb zu konstruieren und sich vom ideologischen Gegner abzugrenzen. Solche Abgrenzungen haben nicht nur die Darstellung des Kalten Krieges als ideologische, aber friedliche Auseinandersetzung in der bundesdeutschen Gesellschaft maßgeblich geprägt. Darüber wurden auch Grenzziehungen zwischen Freiheit und Unterdrückung, zwischen West und Ost, ja sogar zwischen dem „Abendland“ als zivilisierter Teil Europas und dem ‚unzivilisierten‘, sowjetisch geprägten Teil verhandelt.^37^ So könnte auch ein wirtschafts- und unternehmensgeschichtlicher Ansatz an politische und gesellschaftliche Prozesse rückgebunden werden. Hier wäre der Kalte Krieg als ein „politisches Projekt“, wie Philipp Sarasin unlängst angemahnt hat, zu untersuchen, ohne dass er als „selbstverständliche Epochenbezeichnung“ vorausgesetzt würde.^38^ Die Marketingstrategen der bundesdeutschen Rüstungsindustrie, aber auch die Journalist_innen und Vertreter_innen der sozialen Bewegungen, haben nämlich, wie aus den Ausführungen van der Kerkhofs abzuleiten ist, ihr Scherflein zur diskursiven Verteidigung des Abendlandes ebenso beigetragen wie zu den Aushandlungen um die Westernisierung der Bundesrepublik im Sinne einer gemeinsamen europäisch-transatlantischen Werteordnung.^39^ Wie sie damit gesellschaftliche Diskurse veränderten, außenpolitisches Handeln beeinflussten und sowjetische Reaktionen anstießen, bleibt gleichermaßen noch zu untersuchen wie die diskursiven und militärischen Verlagerungen des Konflikts in andere Weltregionen, wozu wiederum der westdeutsche Waffenexport beigesteuert hat. Ähnliches gilt auch für die Frage, wie das Ende des Kalten Krieges, das van de Kerkhof in einer deutschen Perspektive im Text auf 1989 und im Untertitel auf 1990 legt, von den hier im Fokus stehenden Akteur_innen verhandelt wurde und wie sich durch die wahrgenommene Zäsur ihre Praktiken und Deutungen änderten.

## Neue kultur- und verflechtungsgeschichtliche Perspektiven auf den Kalten Krieg

Aus dem ausgelaufenen Münsteraner Sonderforschungsbereich „Kulturen des Entscheidens“ ist der Sammelband „Politisches Entscheiden im Kalten Krieg. Orte, Praktiken und Ressourcen in Ost und West“ hervorgegangen.^40^ Die beiden Herausgeber Thomas Großbölting und Stefan Lehr nähern sich politischen Entscheidungen, in dem sie die Art und Weise in den Mittelpunkt rücken, wie diese sozial und kommunikativ zustande gekommen sind. In Abgrenzung zu einem klassischen politikgeschichtlichen Zugriff arbeiten sie dem Forschungsprogramm des Sonderforschungsbereichs entsprechend mit dem Begriff der „Kultur des Entscheidens“ (S. 8) beziehungsweise im „Modus des Entscheidens“ (S. 9). Nach Luhmann manifestiert sich Politik im Entscheiden. Die oft unhinterfragten Voraussetzungen des Entscheidens sind vielfältig, weil „sich Weltsichten, Ideologien, politische Überzeugungen und Programme von einzelnen Akteuren und Gruppen, aber auch situative Zwänge, zeitliche Dynamiken und langfristige Prozesse in unterschiedlichen Systemen in die Praxis von Politik umsetzen“ (ebd.). Sie sollen anhand von Praktiken, der diskursiven und symbolischen Legitimation in der spezifischen Konstellation des Kalten Krieges für ost- und westeuropäische Gesellschaften analysiert werden. Entscheiden im Kalten Krieg sei im Bereich der internationalen Politik, so betonen die Herausgeber einleitend, weitreichender gewesen als je zuvor, weil eine falsche Entscheidung die Welt hätte nuklear zerstören können (ebd.). Diese, auf die Supermächte zugespitzte Perspektive sollen die Beiträge des Sammelbandes erweitern, indem sie die soziokulturellen und politischen Kontexte des Entscheidens untersuchen. Dabei ist nicht nur das ‚Was‘ und ‚Wie‘ interessant, sondern auch, was historisch jeweils überhaupt als entscheidungsbedürftig und entscheidbar wahrgenommen wurde (S. 13). Diese Prozesse sollen mit Blick auf Parallelen und Unterschiede in Ost und West vergleichend betrachtet werden (S. 16). Diesen vielversprechenden und sehr verdienstvollen Anspruch löst der Band jedoch nicht ein, da die Beiträge keinen vergleichenden Zugriff anbieten, sondern sich jeweils mit der Sowjetunion, der ČSSR oder der DDR stellvertretend für ‚den Osten‘ oder mit der Bundesrepublik für ‚den Westen‘ beschäftigen. Weiter betonen die Herausgeber zwar zu Recht, dass sich der Kalte Krieg in nahezu alle Bereiche der Gesellschaft, Kultur, Politik und Wirtschaft einschrieb, sodass die neuere Forschung mittlerweile an einer Gesellschaftsgeschichte des Kalten Krieges arbeite. Doch auch diese Perspektive spiegeln die hier versammelten Beiträge, wenn überhaupt, nur am Rande wider. Der Titel und teilweise auch die Einleitung des Bandes wecken folglich falsche Erwartungen an neue gesellschafts- und kulturgeschichtliche Ergebnisse zum Kalten Krieg als zeitgenössisches Ordnungsmuster: Der Kalte Krieg ist hier eine reine Epochenbezeichnung, konzeptionell spielt er so gut wie keine Rolle. Eine Ausnahme stellt in dieser Hinsicht der letzte Beitrag des Bandes dar, der als einziger auf Englisch verfasst ist. Die Autorin S. M. Amadae analysiert vier neuere Studien zur Verwissenschaftlichung der Entscheidungstheorien im Kalten Krieg. Ihr Interesse gilt der Rolle der Spieltheorie und der *rational-choice*-Theorie als Grundlage für *per se* unsicheres politisch-militärisches Entscheiden angesichts der drohenden nuklearen Zerstörung der Welt. Hier bildet die dem Kalten Krieg inhärente Logik der atomaren Abschreckung zumindest einen zentralen Bezugspunkt, wenn der Beitrag an sich auch keinen geschichtswissenschaftlichen, sondern eher wissenschaftsphilosophischen Zugang wählt. Amadae kommt zu dem Schluss, dass die zeitgenössischen Wissenschaftler_innen die mithilfe von Computersystemen kontrollierte Atombombe als notwendiges Risiko akzeptiert hätten, um das – wie auch immer definierte – Projekt der Aufklärung und Moderne zu retten. Der Preis dieser Rationalität war die Möglichkeit der Ausrottung der Menschheit und habe damit jeglicher moralischer Einbettung entbehrt (S. 249–271).

Der Beitrag von Matthias Völkel zur „Kybernetik als Ressource des politischen Entscheidens in der Sowjetunion“ wirft über einen wissenschaftlichen Transfer eine verflechtungsgeschichtliche Perspektive auf den Kalten Krieg. Die Kybernetik wurde zunächst im Westen entwickelt und verfolgte dort andere Zielsetzungen, indem mit ihr gesellschaftliche Prozesse präziser gesteuert werden sollten. In der Sowjetunion erlangte sie nie denselben Stellenwert, geschweige denn, dass sie politische Entscheidungen strukturell oder inhaltlich tatsächlich verändert hätte. Vielmehr zeichnet Völkel nach, wie diese Theorie der *governance* marxistische Grundannahmen über gesellschaftliche Entwicklungen und damit das Machtmonopol der Kommunistischen Partei infrage stellte. Der spezifische sowjetische ideologische Kontext verhinderte damit einen erfolgreichen Transfer. Stattdessen wurden kybernetische Methoden in volks- und betriebswirtschaftlichen sowie juristischen Bereichen adaptiert, um bekannte Prozesse zu verbessern (S. 165–183).

Die übrigen Beiträge beschäftigen sich aus unterschiedlichen Perspektiven mit Entscheidungsprozessen. Die Autor_innen untersuchen sie in den politischen Machtzentralen und betrachten Personen, die Einfluss auf Entscheidungen hatten oder sie selbst treffen konnten. Neben den Orten, an denen sich die Kulturen des Entscheidens entwickelten, rückt der Band spezifisch die wichtige Frage in den Blick, inwieweit Experten Einfluss auf politische Entscheidungsprozesse gewannen und so die Entscheidungskulturen veränderten.

Die ersten beiden Beiträge von Gabriele Metzler und Stephan Merl tun dies in einer längeren Perspektive für die alte Bundesrepublik und die Sowjetunion. Während Metzler den Wandel der westdeutschen Entscheidungskultur erhellend anhand des Narrativs der Kanzlerdemokratie seit der Adenauer-Ära nachzeichnet, rückt Merl das Politbüro der KPdSU als Machtzentrum in den Mittelpunkt. Auf verschiedenen Ebenen arbeitet er überzeugend heraus, wie sich seit der Revolution von 1917 eine sowjetische spezifische Entscheidungskultur entwickelte. Sie zeichnete sich dadurch aus, dass persönliche Netzwerke, die um den jeweiligen Diktator – in Merls Diktion als „Rolle“ zu verstehen – kreisten, grundsätzlich über institutionellen Rationalitäten standen. Zentral war, dass der Machterhalt des Diktators und der Partei stets wichtiger waren als etwaige sachorientierte Problemlösungen. Diskussion, Aushandlungen und Zustimmung wurden in Versammlungsöffentlichkeiten inszeniert, die öffentliche Kommunikation streng kontrolliert, wobei tatsächliche Funktionsmechanismen, zum Beispiel der Kommandowirtschaft, tabuisiert wurden.

Auch die übrigen Beiträge des Bandes sind für sich genommen instruktiv, ohne dass sie alle an dieser Stelle im Einzelnen besprochen werden, da der Kalte Krieg hier keine Rolle spielt. Sie eröffnen aber, soviel ist festzuhalten, neue Perspektiven auf scheinbar Bekanntes, wie zum Beispiel Stefan Lehr, der das Präsidium des Zentralkomitees (ZK) der KPČ in den Mittelpunkt stellt, um Entscheidungskulturen der ČSSR zu analysieren. Lehr zeichnet einleuchtend nach, wie Entscheidungen bereits im Vorfeld einer Präsidiumssitzung getroffen, dann aber in der Sitzung mit scheinbar ergebnisoffenen Diskussionen inszeniert und legitimiert wurden. Die vorherigen Aushandlungen fanden hierarchisch zwischen dem Generalsekretär, den ZK-Abteilungen und untergeordneten Parteiinstanzen statt. Um Interessen durchzusetzen, waren, wie es für sowjetische politische Systeme typisch war, die persönlichen Netzwerke entscheidend.

Svenja Schnepel analysiert in ihrem ersten Beitrag in diesem Band, wie die Entscheidungsprozesse im Bundeskanzleramt unter den Kanzlern Ludwig Erhard, Kurt Georg Kiesinger und Willy Brandt zunehmend durch die gesellschaftspolitischen Diskurse der 1960er Jahre geprägt wurden. Um Arbeitsabläufe zu optimieren, wurden Wissenschaftler hinzugezogen. So sollte unter anderem mithilfe der neuen Elektronischen Datenverarbeitung Kommunikation und Planung verbessert werden, indem die unterschiedlichen Verwaltungsebenen leichter an den Entscheidungen partizipieren konnten. Während hier der Kalte Krieg als Deutungsrahmen wenig überraschend keinerlei Bedeutung hat, hätte es in ihrem zweiten Aufsatz allerdings nahegelegen, ihn konzeptionell einzubeziehen. Allerdings dient er Schnepel in ihrer Betrachtung über die mögliche atomare Aufrüstung der Bundeswehr lediglich als ein zeitgenössisch scheinbar feststehender Deutungsrahmen, ohne dass sie seinen Einfluss auf die Diskurse und ihn damit als Faktor des Entscheidens historisieren würde.

Zu einer Kulturgeschichte des Entscheidens in der Bundesrepublik, der DDR, der Sowjetunion und der ČSSR trägt der Band eine Menge bei und ergänzt die klassischen politikgeschichtlichen Erkenntnisse produktiv. Dass Titel und Einleitung andere Erwartungen wecken, ist den Autor_innen nicht anzulasten und schmälert ihre Leistung nicht. Dennoch ist die Chance vergeben worden, Transfers, Verflechtungen und Abgrenzungen zu untersuchen, die nicht zuletzt durch das gegenseitige Beobachten, vor allem aber durch wechselseitige Abhängigkeiten in der Systemkonkurrenz entstanden sind.

Diesen wechselseitigen Beziehungen trägt hingegen der von Hélène Miard-Delacroix und Andreas Wirsching herausgegebene Sammelband „Emotionen und internationale Beziehungen im Kalten Krieg“ stärker Rechnung.^41^ Mit einem emotionsgeschichtlichen Zugriff spiegeln die Beiträge in zumeist innovativer Weise den Kalten Krieg als Deutungsrahmen für innen- und außenpolitische Phänomene. In fünf Sektionen diskutieren sie, inwiefern der Systemwettbewerb mit seinen binären Ordnungsmustern auf spezifischen Emotionsregimen basierte, diese hervorbrachte und veränderte. Wenn Gefühle als soziale Konstruktionen von Wirklichkeit verstanden werden und sie auf der Basis eines gemeinsamen Wertesystem quasi erlernt und angeeignet werden können, lässt sich ihre zeitliche Bedingtheit untersuchen. Welche Emotionen waren demnach spezifisch für den Kalten Krieg als Epoche, welche kennzeichneten in dieser Zeit persönliche Beziehungen zwischen Politiker_innen oder welche Emotionsstrukturen rahmten die internationalen Beziehungen? Diese Fragen deuten an, dass sich Emotionen in unterschiedlichen Handlungsräumen und Akteursgruppen beobachten lassen. Einige Autor_innen erhellen zum Beispiel, wie politisch Handelnde mit emotionaler Verbindlichkeit kalkulierten, wie um Vertrauen in informellen Gesprächen und offiziellen diplomatischen Verhandlungen, aber auch gegenüber der eigenen Gesellschaft gerungen wurde. So betrachtet zum Beispiel Jessica Gienow-Hecht die Annäherung zwischen Michail S. Gorbačev und Ronald Reagan unter der Maßgabe der Vertrauensbildung. Vertrauen wird hier im Sinne einer persönlichen Empfindung Reagans gegenüber dem sowjetischen Generalsekretär der KPdSU als ein entscheidendes Momentum politischen Handelns eingeordnet. Reagan baute so einen Erwartungshorizont auf, der rückblickend vermessen werden kann. Allgemein ist zu fragen, warum und in welchen Situationen Politiker_innen auf Vertrauen als Voraussetzung der diplomatischen Kommunikation setzten. Dann ist von Interesse, inwieweit die damit verbundenen Erwartungen in Erfüllung gingen? Reagans Entscheidung, Gorbačev zu vertrauen, führte, wie Gienow-Hecht argumentiert, auf dem Gipfel von Reykjavík im Oktober 1986 dazu, dass die beiden in informellen Gesprächen eine weitreichende atomare Abrüstung vereinbarten. Auch wenn diese letztlich so nicht umgesetzt worden ist, wirkte die persönliche Annäherung der beiden beim friedlichen Ende des Kalten Krieges mit und veränderte die westliche Sichtweise auf die Sowjetunion. Gienow-Hecht bezieht sich auf den oben bereits erwähnten Archie Brown, um Gorbačevs Agieren einzuordnen. Mit Browns akteursorientiertem Zugang betont die Autorin dessen These vom „human factor“ in den außenpolitischen Beziehungen, der zum Ende des Kalten Krieges maßgeblich beitrug. Die Wechselseitigkeit etwaiger transnationaler Emotionsregime wird hier folglich vor allem aus Reagans Perspektive beleuchtet.

Emotionen zwischen Spitzenpolitiker_innen konnten aber auch eine „schlechte Chemie“ hervorrufen, wie Dominik Geppert in seinem Beitrag über Margaret Thatcher und Helmut Kohl verdeutlicht. Die Gründe für ihren „emotionalen Konfrontationskurs“ (S. 269) reichten von Gender-Stereotypen über unterschiedliche Nations- und Europavorstellungen, divergierende Geschichtsbilder bis hin zu verschiedenen persönlichen Kommunikationsstilen, Diskussionen zu emotionalisieren. Im Ergebnis standen diese Divergenzen einem gemeinsamen westlich-transnationalen Emotionsregime entgegen. Dennoch bleibt die Frage, wie nachhaltig sich diese persönlichen Beziehungen auswirkten.

Neben dem Blick auf die handlungsleitenden Emotionen herausragender Personen untersuchen einige Beiträge „emotional communities“. Diese bildeten sich mit gemeinsamen kommunikativen Codes und emotionalen Normen aus. Joachim Scholtyseck zeigt, wie sich die Neue Linke für die Dekolonisierung des Globalen Südens begeisterte und den Ost-West-Konflikt als Deutungsrahmen für politische Utopien, Revolution und Befreiung transferierten. Empörung und Unterstützung für die Unterdrückten der sogenannten ‚Dritten Welt‘ schuf ein spezifisches Emotionsregime, das den Teilhabenden ein Gefühl von Glück und Stolz vermittelte. Martin Schulze Wessel, um einen weiteren der vielen spannenden Beiträge anzusprechen, analysiert, wie sich unter den binären Kommunikationsbedingungen des Kalten Krieges auch die politischen Blöcke als „emotional communities“ auffassen lassen, die sich über bestimmte Emotionssemantiken definierten. Anhand des Konflikts zwischen der Sowjetunion und der ČSSR um den Prager Frühling 1968 verdeutlicht er, wie die Sowjetunion in ihrem Einflussbereich ideologische Emotionsgemeinschaften mit den sozialistischen Bruderstaaten erzwang. Die Sowjetunion erwartete Dankbarkeit und forderte sie über emotional aufgeladene Begriffe wie Freundschaft und Vertrauen ein. Die sowjetischen Medien vermittelten mit ihnen die sowjetische Machtposition gegenüber den anderen Staaten des Warschauer Paktes an das eigene Publikum, während Leonid Brežnev sie in der persönlichen Kommunikation mit den anderen kommunistischen Parteiführern gezielt einsetzte, um diese emotional mit Vertrauensbekundungen unter Druck zu setzen.

Die neuere Forschung hat bereits überzeugend aufgezeigt, dass Angst angesichts der atomaren Bedrohung im Kalten Krieg eine wirkmächtige Emotion war, die bis in den Alltag der Menschen reichte und emotionale Gemeinschaften wie die Anti-Atomkraftbewegung begründen konnte.^42^ Diesen emotionsgeschichtlichen Zugriff auf den Kalten Krieg erweitert dieser Sammelband und beleuchtet viele bereits bekannte, eher politikgeschichtliche Aspekte von einer neuen Seite. Zugleich lädt er dazu ein, die gesellschaftliche Ebene jenseits der politischen Eliten deutlich stärker in den Blick zu nehmen, um auch die alltäglichen Emotionsregime der ‚normalen‘ Menschen zu untersuchen. Ebenso sollten die für den Kalten Krieg spezifischen Emotionsregime noch stärker in ihrer tatsächlichen wechselseitigen und transnationalen Wirkung ausgeleuchtet werden. So können gesellschaftliche Verflechtungen über den Eisernen Vorhang hinweg erkundet werden, die zu Verständigung, Annäherung und der Überwindung der binären Denkmuster beigetragen haben könnten.

Solche Verflechtungen nehmen zwei Dissertationen auf je eigene Art in innovativer Weise in den Blick, indem transnationale Perspektiven die Untersuchungen leiten. Eine kultur- und verflechtungsgeschichtliche Pionierstudie zum Kalten Krieg ist die in Tübingen entstandene Dissertation von Sonja Großmann zu den sogenannten Freundschaftsgesellschaften mit der Sowjetunion in der Bundesrepublik, Frankreich und Großbritannien.^43^ Die Autorin untersucht diese Vereinigungen, die im Westen Kontakte für kulturellen Austausch aufbauten, als transnationale Akteure, die über den Eisernen Vorhang hinweg kommunizierten. Die Freundschaftsgesellschaften knüpften an eine sowjetische Strategie aus der Zwischenkriegszeit an, als ab 1925 die Allunionsgesellschaft für kulturelle Beziehungen mit dem Ausland (VOKS) im Ausland ein positives Bild der jungen Sowjetunion vermitteln sollte. Sie lud interessierte Ausländer_innen – oft sogenannte *fellow traveller* – ein, die Sowjetunion unter Anleitung der VOKS zu bereisen, gründete aber auch schon Unterstützungsgesellschaften im Ausland. Im Kontext des friedlichen Systemwettbewerbs wurden nach Stalins Tod die Strategien der Kulturdiplomatie und der *soft power* immer wichtiger. Auch die Sowjetunion versuchte nun, aktiv um die ‚Herzen und Köpfe‘ der Menschen zu konkurrieren. Sie lud junge Menschen 1957 zu den Weltfestspielen der Jugend und Studenten in die Sowjetunion ein, entsandte Künstler_innen und Sportler_innen in den Westen, ließ Pariser Modeschöpfer in Moskau präsentieren, amerikanische Musiker Konzerte geben oder gründete 1960 die Universität der Völkerfreundschaft für Studierende aus Afrika, Asien und Lateinamerika. Wie sich die sowjetische Kulturdiplomatie in den adressierten Gesellschaften und auch in der Sowjetunion auswirkte, welche Kontakte, Verflechtungen und Abgrenzungen so entstanden, ist erst ansatzweise untersucht worden. Großmann unternimmt also einen wichtigen Schritt, diese Lücke zu schließen, indem sie fragt, welche Wirkungen die Freundschaftsgesellschaften im Westen entfalten konnten und wer sich aus welchen Motiven in ihnen engagierte (S. 4). In Westeuropa und nicht zuletzt in der Bundesrepublik und Großbritannien, wo der Kommunismus – im Gegensatz zu Frankreich und Italien mit starken kommunistischen Parteien – ein wirkmächtiges Feindbild war, wurden die Freundschaftsgesellschaften häufig als verlängerter Arm Moskaus und „nützliche Idioten“ (S. 336) misstrauisch beäugt. Angesichts dieser unterschiedlichen innenpolitischen Konstellationen erschließt sich unmittelbar das große Potenzial, das die Studie für eine europäisch-transnationale Geschichte des Kalten Krieges entfaltet. Denn Großmann untersucht vergleichend die Handlungsspielräume und Interessen der Mitglieder der Freundschaftsgesellschaften ebenso wie die sowjetischen Strategien im Umgang mit den jeweiligen innen- und außenpolitischen Konstellationen. Auf diese Weise arbeitet die Autorin anschaulich heraus, wie die Freundschaftsgesellschaften soziopolitisch verankert waren und mit Beginn des Tauwetters nach Stalins Tod aktiv das Bild der Sowjetunion im Westen mitkonstruierten. Großmann schreibt dezidiert keine Geschichte einer ideologischen Niederlage. Im Gegenteil verdeutlicht sie überzeugend, mit welchen unterschiedlichen Interessen sich die Menschen in den westlichen Freundschaftsgesellschaften für die Sowjetunion engagierten und so trotz ideologischer Differenzen zur Entspannung und zu gegenseitigem Verständnis beitrugen. Dabei analysiert die Verfasserin kenntnisreich die jeweiligen innenpolitischen Konstellationen, in denen die Freundschaftsgesellschaften agierten, und zeigt, wie Moskau zum Teil sehr flexibel auf die verschiedenen nationalen Interessenlagen reagierte. Tatsächlich spielten zum Beispiel DKP-Aktivist_innen in den westdeutschen Freundschaftsgesellschaften aus taktischen Gründen kaum eine Rolle, hätten sie doch – auch aus Sicht des sowjetischen Außenministeriums – die Glaubwürdigkeit der regionalen Organisationen eher untergraben. Der „British-Soviet Friendship Society“ gelang es hingegen nur mit Schwierigkeiten, Mitglieder jenseits kommunistischer Kreise anzuziehen, wobei sie insbesondere *Labour*-Anhänger erst vereinzelt ab Mitte der 1970er Jahre gewinnen konnte, als die antisowjetische Politik der Partei bei einigen Anhänger_innen Unzufriedenheit hervorrief. In Frankreich war die Situation anders. Hier beteiligten sich Mitglieder des *Parti communiste* rege an der „Association France-URSS“, während die Mehrzahl der Mitglieder der westdeutschen Freundschaftsgesellschaften dem sozialdemokratischen und liberalen Lager angehörten. Ab den 1960er Jahren organisierte die französische Freundschaftsgesellschaft Reisen in die Sowjetunion, die sie quasi kommerziell anbot, und zog mit den zahlreichen Kulturveranstaltungen auch Mitglieder aus sozialistischen und gaullistischen Kreisen an. Zudem hegten viele Franzosen aus Dankbarkeit für deren Beteiligung am Sieg im Zweiten Weltkrieg Sympathien für die Sowjetunion.

Die Studie löst ihren Anspruch, zu zeigen, wie der Austausch von Personen, Informationen und Ideen durch den Eisernen Vorhang funktionierte, wo die Grenzen verliefen und wie sie sich verschoben, hervorragend ein. Sie zeichnet nach, wie sich die sowjetische Kulturdiplomatie wandelte, wie sie zugleich auch auf die sowjetische Gesellschaft zurückwirkte und letztlich in einem gewissen Rahmen zu einer nicht intendierten Öffnung und Internationalisierung führte. Der Eiserne Vorhang wurde auf diese Weise zu einer „selektiv permeablen Membran“ (S. 30). Die Freundschaftsgesellschaften wirkten nicht nur auf die eigene Gesellschaft, indem sie versuchten, ein positives Bild der Sowjetunion zu vermitteln, sondern auch auf die zwischenstaatlichen Beziehungen, weil sie durch ihre Arbeit friedliche transnationale Beziehungen mit Leben füllten. Dies galt auch, wenn die sowjetischen Akteur_innen letztlich nur nach Maßgabe der Partei und staatlicher Organisationen agieren konnten und Moskau entsprechend auf die Kontakte einwirkte. Durch den Vergleich zwischen der Bundesrepublik, Frankreich und Großbritannien kann die Autorin verdeutlichen, wie unterschiedlich ‚der Westen‘ auf den neuen Generalsekretär Michail S. Gobarčev und die beginnende Umgestaltung der Sowjetunion reagierte. Die Perestrojka und schließlich der Zerfall der Sowjetunion ließen vor allem in Frankreich und Großbritannien viele, wenn nicht sogar die meisten Mitglieder der Freundschaftsgesellschaften geschockt bis desillusioniert und tief enttäuscht zurück, weil ihre politische Utopie gescheitert war. Interessanterweise beobachtet die Autorin auch über den politischen Umbruch hinaus eine zumindest für die westdeutschen Gesellschaften erhebliche Kontinuität unter den Mitgliedern. Stärker als in Frankreich und Großbritannien, wo die personelle Kontinuität geringer war, stand weiterhin die Idee der Völkerverständigung und das Interesse an kulturellem Austausch im Vordergrund. Sie unterstützten die Demokratisierung, boten finanzielle Hilfen und humanitäre Projekte gerade in der Not der turbulenten 1990er Jahre an. Zudem waren ihre Erfahrungen und Kontakte nun auch auf westlicher Seite sehr gefragt, sie galten als Expert_innen für die transnationalen Beziehungen zu Russland (S. 518–520). Schließlich hatten sie dazu beigetragen, dass die Sowjetunion immer weniger als Feindbild wahrgenommen wurde und Sympathien im Westen gewann (S. 524). Die Kulturdiplomatie wirkte dabei allerdings wechselseitig als „graduelle Veränderung des Blickwinkels“, wie Großmann betont (S. 538). Das Ausmaß der Wechselseitigkeit ist eines der zentralen und neuen Erkenntnisse, die die Studie herausarbeitet.

Der Aspekt der Wechselseitigkeit zwischen den politischen Systemen und wirtschaftlichen Abhängigkeiten steht auch in einem weiteren jüngst erschienenen Werk im Mittelpunkt. Die ebenfalls in Tübingen verteidigte Dissertation von Martin Deuerlein beschäftigt sich in einer ideen- und diskursgeschichtlichen Perspektive mit dem Konzept der Interdependenz.^44^ Als Gegenwartsdiagnose war sie im Westen der Schlüsselbegriff einer Epoche, nämlich der langen 1970er Jahre, bevor sie Anfang der 1980er Jahre an Bedeutung verlor und der heute ubiquitäre Begriff der Globalisierung an Deutungsmacht gewann. Eine spezifisch neue Vorstellung von Interdependenz setzte sich in den späten 1960er Jahren durch. Nun löste sich der Begriff von einer normativen Idee des Fortschritts und nahm ebenso Differenzierungen und negative Folgen von globaler Vernetzung wahr. Er bezeichnete vor allem nicht mehr die reine Summe nationaler Entitäten, sondern beleuchtete transnationale Netzwerke, globale Zusammenhänge, die Verflechtung von transnationalen Akteuren, Märkten und Warenströmen. Es waren vor allem westliche Sozialwissenschaftler_innen, die mit diesem Ansatz die internationalen Beziehungen und weltwirtschaftliche Vernetzungen in ein neues Licht rückten. Im Kontext der Entspannungspolitik des Kalten Krieges galten die 1970er Jahre als das „Zeitalter der Interdependenz“, sodass wissenschaftliche Deutungsangebote mit außenpolitischen Interessen zu korrelieren schienen. So wie die Sozialwissenschaften in den 1970er Jahren selbstbewusst beanspruchten, sozioökonomische Entwicklungen nicht nur deuten, sondern wissenschaftlich ‚vernünftig‘ steuern zu können, nahmen Außenpolitiker wie Henry Kissinger und Berater wie Zbigniew Brzeziński an, dass auch die globalen Netzwerke unter Führung der USA bewusst koordiniert werden könnten. Der Kalte Krieg und die binäre Weltordnung galten in der Ära Jimmy Carter nahezu als überholte Konzepte, bis die Administration unter Ronald Reagan wieder geopolitische Interessen und die ideologische Abgrenzung zur Sowjetunion betonte. Mit neoliberalen Wirtschaftsstrategien gewann in den frühen 1980er Jahren dann der Begriff der Globalisierung an Einfluss. Dieser Deutungswandel wurde politisch durch die wiederauflebenden Spannungen und Rüstungskrisen zwischen den Supermächten gerahmt, die globale Verflechtungen und Netzwerke als weniger erstrebenswert erscheinen ließen.

Mit einem vergleichenden Blick auf die USA und die Sowjetunion untersucht Deuerlein die Entwicklung dieser zeitgenössischen Diagnosen über zunehmenden globalen Austausch und über wachsende globale Interaktion, um ‚globales Denken‘ zu historisieren und im Ost-West-Konflikt zu kontextualisieren. Dies gelingt ihm hervorragend. Kenntnisreich und detailliert wirft er mit den amerikanischen und sowjetischen Debatten nicht nur einen Blick auf die wissenschaftlichen Diagnosen, sondern setzt diese in Bezug zum außenpolitischen Handeln der USA und der Sowjetunion. So entsteht ein dichtes Bild der konzeptionellen Dispositionen, mit denen die beiden Supermächte im Systemwettbewerb die Welt wahrnahmen und auf deren Grundlage sie agierten. Anders als in den USA war das Interdependenz-Konzept in der Sowjetunion aus ideologischen Gründen offiziell nicht vertretbar. Allerdings analysierten die sowjetischen Sozialwissenschaftler_innen dieselben Probleme und rezipierten die westliche Forschung, sodass sie mit den Ansätzen vertraut waren. Erst unter Gorbačev stiegen viele von ihnen als außenpolitische Berater und Experten in politisch einflussreiche Positionen auf. Zwar hatte bereits Konstantin U. Černenko Ende 1984 verlautbart, dass ‚globale Probleme‘ wie Umweltverschmutzung und Hunger nur durch Kooperation gelöst werden und zugleich helfen könnten, die amerikanisch-sowjetischen Beziehungen konstruktiv zu entwickeln (S. 329). Doch erst die „neuen Denker“, wie Vadim V. Zagladin und Aleksandr N. Jakovlev, entwickelten mit der Perestrojka auch eine grundlegend neue, reformorientierte Konzeption der internationalen Beziehungen. Die Sowjetunion wollte nun kooperativ globale Probleme ohne ideologische Einflussnahme lösen, während Gorbačev Europa als „gemeinsames Haus“ ausrief. Nach dem Zerfall der Sowjetunion und dem scheinbaren Ende des Kalten Krieges diversifizierten sich die politischen Deutungen der transnationalen Beziehungen zusehends, während die Interdependenz auch als außenpolitisches Konzept nicht mehr anschlussfähig erschien. Indem Deuerlein die Diskurse in den USA und der Sowjetunion nachzeichnet und aufeinander bezieht, erhellt er die Wechselwirkungen zwischen Politik und Sozialwissenschaften auf der einen Seite und ihre Einflüsse auf die binäre Denkstruktur des Kalten Krieges auf der anderen Seite. Dabei erwies sich das politische System aber durchaus auf beiden Seiten des Eisernen Vorhangs als maßgebend für die Deutungsmuster.

## Fazit

Nicht erst Russlands Krieg gegen die Ukraine zeigt, dass Ost-West-Feindbilder nicht nur leicht zu reaktivieren, sondern sogar wieder handlungsleitend sind. Der Krieg und die vorhergehenden Dissonanzen unterstreichen, dass der Ost-West-Konflikt länger andauert(e) als die Phase des Kalten Krieges. Dabei wären seine politischen und kulturellen (Dis‑)Kontinuitäten, die einige Studien ansprechen, in einer längeren Perspektive für das 20. und 21. Jahrhundert zu untersuchen. Was zeichnete ihn nach 1917 aus? Inwiefern verblasste er nach 1991 zunächst, ohne aus der Welt zu sein? Auch die nach dem Zweiten Weltkrieg so gängige Metapher des Eisernen Vorhangs stammte schon aus den späten 1920er Jahren. Angesichts dieser Kontinuitäten leuchtet es ein, dass einige der hier vorgestellten Bücher, wie das von Kyong-Min Son oder Guido Thiemeyer, bereits in der Zwischenkriegszeit einsetzen, um die Genese des Kalten Krieges zu erklären. Doch auch für die Nachkriegszeit wird der Beginn des Kalten Krieges nicht mehr so eindeutig festgelegt, wie es lange galt. In einer globalen Perspektive, die Thomas K. Robb und James Gill in „Divided Allies“ auf den asiatisch-pazifischen Raum zum Ende des Zweiten Weltkrieges werfen, stellt sich der Übergang zum Kalten Krieg früher und fließend dar. Für das Ende des Kalten Krieges erscheinen die Einschnitte 1989 mit dem Fall der Berliner Mauer und 1991 mit dem Zerfall der Sowjetunion wirkmächtig und bis zum russischen Einmarsch in die Ukraine fast eindeutig. Dennoch ist auch das eine Frage der Perspektive, wie erste Studien zeigen. So hat Jan Hansen nachgezeichnet, wie der Kalte Krieg im Kontext des NATO-Doppelbeschlusses für weite Teile der deutschen Sozialdemokratie schon um 1980 als Ordnungskategorie deutlich an Erklärungskraft verlor. Ohne dass alle Sozialdemokraten diesen Umschwung mittrugen, sieht Hansen die Entwicklung als Spiegel der Gesellschaft.^45^ Zwar muss sich die SPD aktuell genau diesen Fragen wieder stellen. Doch inwieweit der Perspektivwechsel jenseits der linksliberalen Anhänger der Neuen Ostpolitik und der Friedensbewegung in die bundesdeutsche Gesellschaft strahlte und das Deutungsmuster des Kalten Krieges als Anachronismus erscheinen ließ, bleibt noch zu erforschen. Gleiches gilt für die Frage, wie sich solche Diskursmuster in den anderen westeuropäisch-transatlantischen Gesellschaften verschoben. Beides sind angesichts der aktuellen Konfrontation plötzlich wieder überaus relevante Perspektiven.

Die hier besprochenen Studien interessieren sich für eher politikgeschichtliche Aspekte, die sich mit dem Kalten Krieg als Zäsur verbanden. Alle unterstreichen, wie relevant, vielfältig und dynamisch das Forschungsfeld zum Kalten Krieg ist. Zwar erscheinen nach wie vor Studien, die, wie die Darstellungen von Archie Brown und Stephan Kieninger, einen klassischen politikgeschichtlichen Ansatz verfolgen und eher nur Nuancen zu bekannten Aspekten der Politik- und Diplomatiegeschichte des Kalten Krieges hinzufügen. Dafür zeigen die Untersuchungen von Thomas K. Robb und James Gill zu „Divided Allies“ und von Roberto Cantoni über „Oil Exploration, Diplomacy, and Security in the Early Cold War“, dass auch politik- und diplomatiegeschichtlich noch zahlreiche offene Fragen systematisch beantwortet werden können, wenn sie – wie in diesen Fällen – mit globalen, transnationalen und/oder wirtschaftshistorischen Ansätzen verbunden werden und den Kalten Krieg als Ordnungsschema reflektieren. Außerdem haben neben Robb und Gill bereits andere neuere Studien gezeigt, dass es aus lokaler oder (welt-)regionaler Sicht konkurrierende Ordnungsvorstellungen zum Kalten Krieg gab, die quer zu den klassischen Phasen der Entspannung und Krise lagen und die entsprechend auch auf das Handeln der Supermächte zurückwirken konnten. In diesem Sinne agierte die Sowjetunion häufiger pragmatisch denn ideologisch in ihren Beziehungen zum *Global South*.^46^

Insofern ist eine globale beziehungsweise transnationale Perspektive, wie sie diese Autoren, aber auch die kulturgeschichtlichen Untersuchungen von Sonja Großmann und Martin Deuerlein einnehmen, notwendig, um die Binarität des Kalten Krieges zu dekonstruieren und scheinbare Gewissheiten nicht zuletzt über die Haltung einzelner westlicher Länder zur Sowjetunion zu hinterfragen. Offenbar ließen sich andere Deutungsrahmen, wie die Interdependenz, die Konvergenz der Systeme, der Globalisierung, Liberalisierung oder Dekolonisierung, variabel mit dem narrativen Code des Kalten Krieges verbinden.^47^

Mit solchen erweiterten Perspektiven differenziert sich das Bild nicht nur hinsichtlich im Westen durchaus divergierender außenpolitischer Interessen deutlich, sondern auch mit Blick auf verschiedene innergesellschaftliche Positionen im Westen zur Sowjetunion. Dabei hat Roberto Cantoni eindrucksvoll den Stellenwert wirtschaftlicher Interessen für außenpolitisches Handeln aufgezeigt, die ideologische Vorbehalte auch im Westen überlagern konnten, zumal, wenn es eine starke kommunistische Partei wie in Frankreich und Italien gab. Dennoch mangelt es an wirtschaftsgeschichtlichen Arbeiten, die den Kalten Krieg als handlungsleitenden Rahmen einbeziehen. Weiter fehlen Studien, die die wirtschaftlichen Verflechtungen, die von der Sowjetunion selbst und von den Staaten ihrer Einflusszone zum Globalen Süden und zum Westen aufgebaut worden sind, stärker in den Blick rücken. Dazu gehört auch, die Konkurrenz zwischen China und der Sowjetunion als Akteure in der Dekolonisation eingehender als bisher zu betrachten, um den Kalten Krieg geopolitisch und ideologisch weiter zu dezentrieren und multiperspektivisch auch den Interessen und der *agency* der sich dekolonisierenden Staaten stärker Rechnung zu tragen.

Generell zeigen die hier besprochenen Untersuchungen, dass Osteuropa im Allgemeinen und die Sowjetunion als Supermacht im Besonderen zumeist keinen gleichrangigen analytischen Stellenwert haben. Oft werden ost(mittel)europäische Akteur_innen nur in den Wahrnehmungen und Handlungen westlicher Akteur_innen gespiegelt. Studien, die tatsächlich Verflechtungen, Transfers und Abgrenzungen, die den Eisernen Vorhang überbrückten, gleichwertig untersuchen, sind noch zu selten. Die Dissertationen von Großmann und Deuerlein stellen nach wie vor sehr begrüßenswerte Ausnahmen im Forschungsfeld dar, sobald die Ebene der ‚hohen‘ Diplomatie verlassen wird. Dabei verdeutlicht gerade die Arbeit von Sonja Großmann, die zum einen gesellschaftliche Akteur_innen und ihre Praktiken in den Mittelpunkt rückt, das Potenzial eines solchen verflechtungsgeschichtlichen Ansatzes. Nur so lassen sich Kontakte und Prozesse der Verständigung untersuchen, die die Entspannungspolitik stützten und in die Mitte der Gesellschaft tragen konnten. Ebenso lassen sich nur so innergesellschaftliche Differenzen über die Haltung zur Sowjetunion verfolgen. Zum anderen kann Großmann mit ihrem Ansatz nachzeichnen, inwiefern die Sowjetunion Strategien der *soft power* und *cultural diplomacy* einsetzte und welche Wirkung diese auf welche westlichen Akteur_innen überhaupt entwickeln konnten. Gleichzeitig ist auf diesem Wege zu erkennen, inwiefern Kontakte über den Eisernen Vorhang hinweg – so staatlich kontrolliert sie auch waren – zu einer Öffnung oder gar zu einem innergesellschaftlichen Wandel in den ost(mittel)europäischen Staaten führen konnten.

Zu den weißen Flecken, die künftige Forschungsprojekte angehen sollten, gehört auch eine Mediengeschichte des Kalten Krieges, die in den hier vorgestellten Büchern keine Rolle spielt. Nach ersten Arbeiten zu den Auslandsradios ist die mediale Dimension des *cultural cold war* in den Hintergrund getreten.^48^ Die Frage aber, wie Medien Wahrnehmungen und Deutungen rahmten, wie Journalist_innen die Wahrnehmungs- und Kommunikationsprozesse, die den Eisernen Vorhang überbrückten, prägten, ist nicht zuletzt angesichts heutiger medialer Auseinandersetzungen wieder überaus relevant. Nicht nur im Kalten Krieg produzierten die wechselseitigen medialen Beobachtungen konkurrierende Vorstellungen über den jeweils ‚Anderen‘. Gleichzeitig stellten sie trotz der Systemkonkurrenz die Relationen und Verflechtungen her, in denen gesellschaftliche Selbst- und Fremdbeschreibungen kommuniziert wurden.^49^ Auch hier weist ein verflechtungsgeschichtlicher Ansatz den Weg, die binäre Logik des Kalten Krieges aufzubrechen und ihn als transnationale Geschichte von Konvergenzen, Zirkulationen und Abgrenzungen von Akteur_innen, Praktiken, Ideen und Deutungen zu erzählen. Als politisch-ideologische Weltordnung, die viele, wenn nicht die meisten Gesellschaften auf der Welt beeinflusst hat, fordert der Kalte Krieg eine transnationale und auch globale Perspektive geradezu ein.

## Auswahlbibliografie

- Brown, Archie: The Human Factor. Gorbachev, Reagan, and Thatcher, and the End of the Cold War, 512 S., Oxford UP, Oxford u. a. 2020.
- Cantoni, Roberto: Oil Exploration, Diplomacy, and Security in the Early Cold War. The Enemy Underground, 308 S., Routledge, London/New York 2017.
- Deuerlein, Martin: Das Zeitalter der Interdependenz. Globales Denken und internationale Politik in den langen 1970er Jahren (Moderne Zeit. Neue Forschungen zur Gesellschafts- und Kulturgeschichte des 19. und 20. Jahrhunderts, Bd. 31), 500 S., Wallstein, Göttingen 2020.
- Großbölting, Thomas/Lehr, Stefan (Hrsg.): Politisches Entscheiden im Kalten Krieg. Orte, Praktiken und Ressourcen in Ost und West (Kulturen des Entscheidens, Bd. 2), 273 S., Vandenhoeck & Ruprecht, Göttingen 2020.
- Großmann, Sonja: Falsche Freunde im Kalten Krieg? Sowjetische Freundschaftsgesellschaften in Westeuropa als Instrumente und Akteure der Cultural Diplomacy (Studien zur Internationalen Geschichte, Bd. 46), 612 S., De Gruyter Oldenbourg, Berlin u. a. 2019.
- Kerkhof, Stefanie van de: Waffen und Sicherheit im Kalten Krieg. Das Marketing der westdeutschen Rüstungsindustrie 1949–1990 (Jahrbuch für Wirtschaftsgeschichte. Beihefte, Bd. 24), 548 S., De Gruyter Oldenbourg, Berlin u. a. 2019.
- Kieninger, Stephan: The Diplomacy of Détente. Cooperative Security Policies from Helmut Schmidt to George Shultz, 236 S., Routledge, London/New York 2018.
- Miard-Delacroix, Hélène/Wirsching, Andreas (Hrsg.): Emotionen und internationale Beziehungen im Kalten Krieg (Schriften des Historischen Kollegs. Kolloquien, Bd. 104), 430 S., De Gruyter Oldenbourg, Berlin u. a. 2020.
- Robb, Thomas K./Gill, David James: Divided Allies. Strategic Cooperation Against the Communist Threat in the Asia-Pacific During the Early Cold War, 288 S., Cornell UP, Ithaca, NY 2019.
- Son, Kyong-Min: The Eclipse of the Demos. The Cold War and the Crisis of Democracy Before Neoliberalism, 272 S., Kansas UP, Lawrence, KS 2020.
- Thiemeyer, Guido: Totalitarismus und Kalter Krieg (1920–1970), 162 S., Kohlhammer, Stuttgart 2020.

## Footnotes

1 Als kleine Auswahl: Kireev, Maxim: Wie im Kalten Krieg, in: ZEIT online, 14. August 2019, URL: <https://www.zeit.de/politik/ausland/2019-08/russland-skyfall-rakete-radioaktivitaet-wladimir-putin> [Zugriff: 30.09.2020]; Speckmann, Thomas: Russland gibt dem Westen Rätsel auf. Vom alten zum neuen Kalten Krieg, in: Der Tagesspiegel, 30. Juli 2019, URL: <https://www.tagesspiegel.de/kultur/literatur/russland-gibt-dem-westen-raetsel-auf-vom-alten-zum-neuen-kalten-krieg/24589838.html> [Zugriff: 30.09.2020]; Wintour, Patrick/Harding, Luke/Borger, Julian: Cold War 2.0. How Russia and the West Reheated a Historic Struggle, in: The Guardian, 24. Oktober 2016, URL: <https://www.theguardian.com/world/2016/oct/24/cold-war-20-how-russia-and-the-west-reheated-a-historic-struggle> [Zugriff: 30.09.2020]. Auf russischer Seite sprachen Michail Gorbačev wie Dmitrij Medvedev von einem neuen Kalten Krieg: Shuster, Simon: Gorbachev Blames the U.S. for Provoking ‚New Cold War‘, in: TIME, 11. Dezember 2014, URL: https://time.com/3630352/mikhail-gorbachev-vladimir-putin-cold-war/ [Zugriff: 23.10.2020]; o. A.: Russian PM Medvedev says New Cold War is on, in: BBC News, 13. Februar 2016, URL: <https://www.bbc.com/news/world-europe-35569094> [Zugriff: 23.10.2020].

2 Gutschker, Thomas/Löwenstein, Stephan/Schmidt, Friedrich: Sputnik V und die EU. „Instrument eines hybriden Krieges“, in: Frankfurter Allgemeine Zeitung, 9. März 2021, URL: <https://www.faz.net/aktuell/politik/ausland/wie-moskau-die-eu-mit-seinem-impfstoff-sputnik-v-spaltet-17235930.html?premium> [Zugriff: 09.03.2021].

3 Trotz der Ähnlichkeiten hinken die historischen Parallelen zum Kalten Krieg. Ohne das atomare Rüsten spielt die friedenserhaltende Angst vor einem nuklearen Vernichtungsschlag, die den Kalten Krieg charakterisiert hat, eine geringere Rolle. Auch sind die politischen Lager in der multipolaren Welt nach 1991 unübersichtlicher geworden. Insbesondere existiert aber der ideologische Systemkonflikt zwischen Kapitalismus und Sozialismus nicht mehr, was den Einfluss Russlands in der Welt verringert hat.

4 Siehe Schmid, Ulrich: Technologien der Seele. Vom Verfertigen der Wahrheit in der russischen Gegenwartskultur, Suhrkamp, Berlin 2015; Stewart, Susan: Die Konstruktion des Feindbilds Westen im heutigen Russland. Seine Geschichte und seine Funktionen (SWP-Studie S 28), Stiftung Wissenschaft und Politik, Berlin 2008. Ein konkretes Beispiel ist die Kampagne gegen Homosexualität und westliche Liberalität, die sich im Begriff „Gayropa“ verdichtet: Riabov, Oleg/Riabova, Tatiana: The Decline of Gayropa? How Russia Intends to Save the World, in: Eurozine, 5. Februar 2014, URL: <https://www.eurozine.com/the-decline-of-gayropa/> [Zugriff: 20.09.2020].

5 „A Superpower War of Words“, in: Newsweek, 7. November 1983, S. 62.

6 Kaldor, Mary: The Imaginary War. Understanding the East-West Conflict, Blackwell, Oxford 1990; Geyer, Michael: Der kriegerische Blick. Rückblick auf einen noch zu beendenden Krieg, in: Sozialwissenschaftliche Informationen 19 (1990), H. 2, S. 111–117.

7 Niedhart, Gottfried: Der Ost-West-Konflikt. Konfrontation im Kalten Krieg und Stufen der Deeskalation, in: Archiv für Sozialgeschichte 50 (2010), S. 557–594, hier S. 559.

8 So erscheint seit 2018 die Buchreihe „Rethinking the Cold War“ bei De Gruyter Oldenbourg. Sie rückt neben der europäischen Dimension auch die globale in den Blick und interessiert sich besonders für transnationale Akteure, Verflechtungsprozesse und transkulturelle Begegnungen, die den Eisernen Vorhang überbrückten. Da ich selbst Herausgeberin dieser Reihe bin, verweise ich hier nur kursorisch auf die Bände.

9 Als kleiner Querschnitt aus der Vielzahl der Titel, die kulturgeschichtlich argumentieren, siehe: Greiner, Bernd/Müller, Christian Th./Walter, Dierk (Hrsg.): Angst im Kalten Krieg, Hamburger Edition, Hamburg 2009; Vowinckel, Annette/Payk, Marcus M./Lindenberger, Thomas (Hrsg.): Cold War Cultures. Perspectives on Eastern and Western European Societies, Berghahn, Oxford/New York 2012; Eugster, David/Marti, Sibylle (Hrsg.): Das Imaginäre des Kalten Krieges. Beiträge zu einer Kulturgeschichte des Ost-West-Konfliktes in Europa, Klartext, Essen 2015; Grant, Matthew/Ziemann, Benjamin (Hrsg.): Understanding the Imaginary War. Culture, Thought and Nuclear Conflict, 1945–90, Manchester UP, Manchester/New York 2016; Reichherzer, Frank/Droit, Emmanuel/Hansen, Jan (Hrsg.): Den Kalten Krieg vermessen. Über Reichweite und Alternativen einer binären Ordnungsvorstellung, De Gruyter Oldenbourg, Berlin u. a. 2018; Nagornaja, Oksana S. u. a. (Hrsg.): Sovetskaja kul’turnaja diplomatija v uslovijach Cholodnoj vojny 1945–1989, Političeskaja ėnciklopedija, Moskau 2018; Christian, Michel/Kott, Sandrine/Matejka, Ondrej (Hrsg.): Planning in Cold War Europe. Competition, Cooperation, Circulations (1950s–1970s), De Gruyter Oldenbourg, Berlin u. a. 2018.

10 Mit globalgeschichtlicher Perspektive vor allem auf staatliche Akteure: Westad, Odd Arne: The Cold War. A World History, Basic Books, New York 2017; Manke, Albert/Březinová, Kateřina (Hrsg.): Kleinstaaten und sekundäre Akteure im Kalten Krieg. Politische, wirtschaftliche, militärische und kulturelle Wechselbeziehungen zwischen Europa und Lateinamerika, transcript, Bielefeld 2016; Friedman, Jeremy: Shadow Cold War. The Sino-Soviet Competition for the Third World, North Carolina UP, Chapel Hill, NC 2015; McMahon, Robert J. (Hrsg.): The Cold War in the Third World, Oxford UP, Oxford u. a. 2013; zur Dekolonisierung: Bracke, Maud Anne/Mark, James: Between Decolonization and the Cold War. Transnational Activism and its Limits in Europe, 1950s–90s, in: Journal of Contemporary History 50 (2015), H. 3, S. 403–417; zur *cultural diplomacy*: Dragostinova, Theodora: The East in the West. Bulgarian Culture in the United States of America During the Global 1970s, in: Journal of Contemporary History 53 (2018), H. 1, S. 212–239.

11 Mit einem politikgeschichtlichen Fokus auf die Sowjetunion zum Beispiel: Mazov, Sergej V.: Cholodnaja vojna. SSSR i Kongolezskij krizis 1960–1964, Russkij Fond Sodejstvija Obrazovaniju i Nauke, Moskau 2015.

12 Miethe, Ingrid u. a.: Globalization of an Educational Idea. Workers’ Faculties in Eastern Germany, Vietnam, Cuba and Mozambique (Rethinking the Cold War, Bd. 7), De Gruyter Oldenbourg, Berlin u. a. 2019; Katsakioris, Constantin: The Lumumba University in Moscow. Higher Education for a Soviet–Third World Alliance, 1960–91, in: Journal of Global History 14 (2019), H. 2, S. 281–300; Rupprecht, Tobias: Soviet Internationalism After Stalin. Interaction and Exchange Between the Soviet Union and Latin America During the Cold War, Cambridge UP, Cambridge 2015.

13 Dies gilt zum Beispiel für Studien über den Versuch, die Einflüsse westlicher Musik abzuwehren: Tsipursky, Gleb: Socialist Fun. Youth, Consumption and State-Sponsored Popular Culture in the Soviet Union, 1945–1970, Pittsburgh UP, Pittsburgh, PA 2016; zur Solidarität gegen einen „kapitalistischen“ Krieg: Mark, James u. a.: ‚We are with You, Vietnam‘. Transnational Solidarities in Socialist Hungary, Poland, and Yugoslavia, in: Journal of Contemporary History 50 (2015), H. 3, S. 439–464.

14 Siehe zu nicht-staatlichen Akteur_innen: Orlov, Igor B./Popov, Aleksej D.: Skvoz’ „železnyj zanaves“. See USSR!: Inostrannye turisty i prizrak potemkinskich dereven’, Izdatel’skij dom Vyšsej školy ėkonomiki, Moskau 2018; Vonnard, Philippe/Sbetti, Nicola/Quin, Grégory (Hrsg.): Beyond Boycotts. Sport During the Cold War in Europe, De Gruyter Oldenbourg, Berlin u. a. 2018; Mikkonen, Simo/Scott-Smith, Giles/Parkkinen, Jari (Hrsg.): Entangled East and West. Cultural Diplomacy and Artistic Interaction During the Cold War, De Gruyter Oldenbourg, Berlin u. a. 2018; Tondera, Benedikt: Reisen auf Sowjetisch. Auslandstourismus unter Chruschtschow und Breschnew 1953–1982 (Forschungen zur osteuropäischen Geschichte, Bd. 85), Harrassowitz, Wiesbaden 2019; Bönker, Kirsten: „Gloom and Embarrassment Over the Landing of the United States Astronauts …“. Die Mondlandung als sowjetisch-amerikanisches Medienereignis, in: Grampp, Sven (Hrsg.): Cold Moon Rising. Die Berichterstattung über die erste bemannte Mondlandung als Globalgeschichte in Zeiten des Kalten Krieges, Springer VS, Wiesbaden 2021, S. 137–168; Fainberg, Dina: Cold War Correspondents. Soviet and American Reporters on the Ideological Frontlines, JHUP, Baltimore, MD/London 2021.

15 Susan R. Reid hat bereits 2010 die Frage nach Konvergenz, Konversion usw. diskutiert: Reid, Susan R.: The Soviet Pavilion at Brussels ’58: Convergence, Conversion, Critical Assimilation, or Transculturation? (CWIHP Working Paper#62), Woodrow Wilson International Center for Scholars, December 2010, URL: <https://www.wilsoncenter.org/publication/the-soviet-pavilion-brussels-58-convergence-conversion-critical-assimilation-or> [Zugriff: 09.03.2021].

16 Westad: Cold War (wie Anm. 10), S. 1.

17 Nagornaja u. a. (Hrsg.): diplomatija (wie Anm. 9); Batjuk, Vladimir I.: Cholodnaja vojna meždu SŠA i SSSR (1945–1991 gg.): očerki istorii, Ves’ Mir, Moskau 2018; Lipkin, Michail A.: Sovetskij Sojuz i integracionnye processy v Evrope. seredina 1940-ch – konec 1960-ch godov, Universitet Dmitrija Požarskogo, Moskau 2016; Egorova, Natalija I./Čubar’jan, Aleksandr O. (Hrsg.): Cholodnaja vojna 1945–1964 gg. Istoričeskaja retrospektiva, Olma-Press, Moskau 2003; Safronov, Vjačeslav P.: SSSR – SŠA – Japonija v gody „cholodnoj vojny“: 1945–1960 gg., Institut Rossijskoj Istorii RAN, Moskau 2003.

18 Naimark, Norman M.: Stalin and the Fate of Europe. Postwar Struggle for Sovereignty, Harvard UP, Cambridge, MA/London 2019.

19 Reynolds, David: From World War to Cold War. Churchill, Roosevelt, and the International History of the 1940s, Oxford UP, Oxford u. a. 2006, S. 249f.; Martin, Katherine Connor: George Orwell and the Origin of the Term ‚Cold War‘, in: OUPblog, 24. Oktober 2015, URL: <https://blog.oup.com/2015/10/george-orwell-cold-war/https://blog.oup.com/2015/10/george-orwell-cold-war/> [Zugriff: 01.10.2020].

20 Die Metapher des Eisernen Vorhangs hat eine längere und facettenreiche Geschichte. Sie ist zum Teil mit anderen Konnotationen schon in der Zwischenkriegszeit verwendet worden, um unter anderem die Grenze zur jungen Sowjetunion zu bezeichnen. Seit Ende 1944 ist sie ein Begriff der Nazi-Propaganda, mit dem Joseph Goebbels die Ergebnisse der Konferenz von Jalta im Februar 1945 diffamierte. Trotzdem ist er dann in den westlichen und auch sowjetischen Sprachgebrauch übergegangen, um ein Informationsdefizit zu bezeichnen und dann ab 1946/47 die Teilung Europas durch eine politische Systemgrenze zu verbildlichen. Medienwirksam platzierte ihn Winston Churchill in diesem Sinne in seiner Rede im März 1946 in Fulton/Missouri, nachdem er ihn schon 1945 mehrfach benutzt hatte. Auch Konrad Adenauer sprach bereits im Juli 1945 in einem Brief an den Journalisten Hans Rörig davon, dass die Sowjetunion einen „eisernen Vorhang“ in Deutschland herunterließe. Siehe: Adenauer, Konrad: Brief Nr. 22, 5. Juli 1945: An Dr. Hans Rörig, Bern, in: Adenauer. Rhöndorfer Ausgabe: Briefe 1945–1947, hrsg. v. Rudolf Morsey/Hans-Peter Schwarz, bearbeitet v. Hans Peter Mensing, Siedler, Berlin 1983, S. 50–53, hier S. 51. Zur Entwicklung der Metapher siehe: Koller, Christian: Der „Eiserne Vorhang“. Zur Genese einer politischen Zentralmetapher in der Epoche des Kalten Krieges, in: Zeitschrift für Geschichtswissenschaft 54 (2006), H. 4, S. 366–384.

21 Robb, Thomas K./Gill, David James: Divided Allies. Strategic Cooperation Against the Communist Threat in the Asia-Pacific During the Early Cold War, Cornell UP, Ithaca, NY 2019.

22 Brown, Archie: The Human Factor. Gorbachev, Reagan, and Thatcher, and the End of the Cold War, Oxford UP, Oxford u. a. 2020.

23 Ders.: The Gorbachev Factor, Oxford UP, Oxford u. a. 1996; ders.: Seven Years that Changed the World. Perestroika in Perspective, Oxford UP, Oxford u. a. 2007.

24 Ders.: Gorbachev Factor (wie Anm. 23).

25 Taubman, William: Gorbachev. His Life and Times, Norton, New York 2017.

26 Als Augenzeugenbericht des ehemaligen amerikanischen Botschafters in Moskau: Matlock, Jack F. Jr.: Reagan and Gorbachev. How the Cold War Ended, Random House, New York 2004; Moore, Charles: Margaret Thatcher. The Authorized Biography, Bd. 1: Not for Turning, Allen Lane, London 2013; Maynard, Christopher: Out of the Shadow. George H. W. Bush and the End of the Cold War, Texas A&M UP, College Station, TX 2008; Pados, John: How the Cold War Ended. Debating and Doing History, Potomac, Dulles, VA 2011; Taubman: Gorbachev (wie Anm. 25).

27 Yurchak, Alexei: Everything Was Forever, Until It Was No More. The Last Soviet Generation, Princeton UP, Princeton, NJ/Oxford 2006.

28 Cantoni, Roberto: Oil Exploration, Diplomacy, and Security in the Early Cold War. The Enemy Underground, Routledge, London/New York 2017.

29 Perović, Jeronim (Hrsg.): Cold War Energy. A Transnational History of Soviet Oil and Gas, Palgrave Macmillan, Basingstoke/New York 2017; Flade, Falk: Energy Infrastructures in the Eastern Bloc. Poland and the Construction of Transnational Electricity, Oil, and Gas Systems, Harrassowitz, Wiesbaden 2017.

30 Bernd Stöver bezeichnet den Kalten Krieg als eine epochale Einheit, für die er sieben Abschnitte identifiziert hat, in denen sich allerdings Phasen der Krise und Entspannung überlappten und gleichzeitig verliefen. Siehe: Stöver, Bernd: Der Kalte Krieg. Geschichte eines radikalen Zeitalters 1947–1991 (durchgesehene und aktualisierte Neuauflage), Beck, München 2017, S. 19f.

31 Kotkin, Stephen: Armageddon Averted. The Soviet Collapse, 1970–2000, Oxford UP, Oxford u. a. 2001, S. 15–17.

32 Sanchez-Sibony, Oscar: Red Globalization. The Political Economy of the Soviet Cold War from Stalin to Khrushchev, Cambridge UP, Cambridge 2014.

33 Rudolph, Karsten: Wirtschaftsdiplomatie im Kalten Krieg. Die Ostpolitik der westdeutschen Großindustrie 1945–1991, Campus, Frankfurt a. M./New York 2004.

34 Kieninger, Stephan: The Diplomacy of Détente. Cooperative Security Policies from Helmut Schmidt to George Shultz, Routledge, London/New York 2018.

35 Kerkhof, Stefanie van de: Waffen und Sicherheit im Kalten Krieg. Das Marketing der westdeutschen Rüstungsindustrie 1949–1990 (Jahrbuch für Wirtschaftsgeschichte. Beihefte, Bd. 24), De Gruyter Oldenbourg, Berlin u. a. 2019.

36 So heißt das Kapitel 3.3 „Neue Marketingstrategien am Ende des Kalten Krieges“.

37 Schildt, Axel: Zwischen Abendland und Amerika. Studien zur westdeutschen Ideenlandschaft der 50er Jahre, Oldenbourg, München 1999.

38 Sarasin, Philipp: Die Grenze des „Abendlandes“ als Diskursmuster im Kalten Krieg. Eine Skizze, in: Eugster, David/Marti, Sibylle (Hrsg.): Das Imaginäre des Kalten Krieges. Beiträge zu einer Kulturgeschichte des Ost-West-Konfliktes in Europa, Klartext, Essen 2015, S. 19–43.

39 Siehe dazu: Doering-Manteuffel, Anselm: Westernisierung. Politisch-ideeller und gesellschaftlicher Wandel in der Bundesrepublik bis zum Ende der 60er Jahre, in: Schildt, Axel/Siegfried, Detlef/Lammers, Karl Christian (Hrsg.): Dynamische Zeiten. Die 60er Jahre in den beiden deutschen Gesellschaften, Christians, Hamburg 2000, S. 311–341.

40 Großbölting, Thomas/Lehr, Stefan (Hrsg.): Politisches Entscheiden im Kalten Krieg. Orte, Praktiken und Ressourcen in Ost und West (Kulturen des Entscheidens, Bd. 2), Vandenhoeck & Ruprecht, Göttingen 2020.

41 Miard-Delacroix, Hélène/Wirsching, Andreas (Hrsg.): Emotionen und internationale Beziehungen im Kalten Krieg (Schriften des Historischen Kollegs. Kolloquien, Bd. 104), De Gruyter Oldenbourg, Berlin u. a. 2020.

42 Siehe z. B. die Beiträge in: Greiner/Müller/Walter (Hrsg.): Angst (wie Anm. 9).

43 Großmann, Sonja: Falsche Freunde im Kalten Krieg. Sowjetische Freundschaftsgesellschaften in Westeuropa als Instrumente und Akteure der Cultural Diplomacy (Studien zur Internationalen Geschichte, Bd. 46), De Gruyter Oldenbourg, Berlin u. a. 2019.

44 Deuerlein, Martin: Das Zeitalter der Interdependenz. Globales Denken und internationale Politik in den langen 1970er Jahren (Moderne Zeit. Neue Forschungen zur Gesellschafts- und Kulturgeschichte des 19. und 20. Jahrhunderts, Bd. 31), Wallstein, Göttingen 2020.

45 Hansen, Jan: Abschied vom Kalten Krieg? Die Sozialdemokraten und der Nachrüstungsstreit (1977–1987), De Gruyter Oldenbourg, Berlin u. a. 2016, S. 2, S. 239–246.

46 Vgl. Sanchez-Sibony: Globalization (wie Anm. 32).

47 Reichherzer, Frank: Mit dem ‚Kalten Krieg‘ experimentieren. Ein Denkanstoß, in: ders./Droit/Hansen (Hrsg.): Den Kalten Krieg vermessen (wie Anm. 9), S. 1–14.

48 Vgl. Badenoch, Alexander/Fickers, Andreas/Henrich-Franke, Christian (Hrsg.): Airy Curtains in the European Ether. Broadcasting and the Cold War, Nomos, Baden-Baden 2013.

49 Vgl. die ersten Arbeiten von Fainberg: Cold War Correspondents (wie Anm. 14); Bönker: Gloom (wie Anm. 14).

